# Cardiovascular Toxicity Associated With Bispecific Antibodies in Hematological Malignancies: A Comprehensive Pharmacovigilance Analysis

**DOI:** 10.1002/ajh.70449

**Published:** 2026-07-24

**Authors:** Malak Munir, Ahmed Sayed, Dae Hyun Lee, Sanam Ghazi, Mustafa M. Houmsse, Saad Badat, Abdul Moeed, Avirup Guha, Daniel Addison, Narendranath Epperla

**Affiliations:** ^1^ Department of Medicine Rochester General Hospital Rochester New York USA; ^2^ Division of Cardiovascular Medicine, Thalheimer Center for Cardio‐Oncology University of Pennsylvania Medicine Philadelphia Pennsylvania USA; ^3^ Cardio‐Oncology Program University of Texas Southwestern Medical Center Dallas Texas USA; ^4^ Division of Cardiology, Department of Medicine Medical College of Georgia at Augusta University Augusta Georgia USA; ^5^ Division of Hematology and Hematologic Malignancies Huntsman Cancer Institute, University of Utah Salt Lake City Utah USA

**Keywords:** bispecific antibodies, cardiovascular toxicity, cytokine release syndrome, FAERS, hematologic malignancies, pharmacovigilance

## Abstract

Cardiovascular adverse events (CVAEs) associated with bispecific T‐cell engaging antibodies (BsAbs) have not been systematically investigated across approved agents. In this disproportionality analysis of FAERS (December 2014–September 2025), reports listing BsAbs as the primary suspected drug (*n* = 7647) were compared with all other drugs in the database (*N* = 7 289 316) and appropriate active comparators. Adjusted reporting odds ratios (aRORs) were estimated using multivariable logistic regression, adjusting for age, sex, cardiovascular comorbidity, disease, and concomitant cardiotoxic medications. Among 7647 BsAb‐associated reports (median age: 60 [36–71] years; 44.6% female), 1408 (18.4%) involved a CVAE and 447 (5.8%) were fatal. BsAbs showed increased reporting of DIC (aROR: 4.35 [3.18–5.96]; *n* = 49), hypotension (1.61 [1.38–1.89]; *n* = 181), and fatal CVAEs (1.63 [1.47–1.80]; *n* = 447). MM‐directed BsAbs were associated with shock (2.09 [1.65–2.66]) and myocarditis (3.68 [1.49–9.10]). Blinatumomab was associated with DIC (4.41 [3.05–6.37]) and hypotension (1.46 [1.18–1.81]). Teclistamab showed the strongest myocarditis signal (5.95 [2.16–16.43]; *n* = 4). Mosunetuzumab showed increased reporting of atrial fibrillation (2.25 [1.15–4.38]) and supraventricular tachycardia (2.21 [1.17–4.15]). CVAEs occurred earlier than non‐CVAEs (median 7 vs. 15 days; *p* < 0.001). The majority of hypotension (56.4%) and heart failure (44.2%) reports occurred independently of both CRS and infection. Case fatality proportions for shock (60.1%), DIC (51.0%), and heart failure (48.1%) exceeded those for CRS (24.3%). BsAb‐associated cardiovascular signals, particularly DIC, hemodynamic events, and fatal CVAEs, varied by agent and target antigen. These findings support the need for cardiovascular‐specific monitoring during therapy.

## 1 Introduction

Bispecific T‐cell engaging antibodies (BsAbs) are a class of immunotherapeutic drugs that direct endogenous T cells to target malignant cells bearing tumor‐associated antigens. These agents have two binding domains, the first targeting CD3 on T cells and another targeting a tumor‐specific antigen (CD19, CD20, BCMA, or GPRC5D) [1].

Since the approval of blinatumomab for B‐cell acute lymphoblastic leukemia (B‐ALL) in 2014, six additional BsAbs have received Food and Drug Administration (FDA) approval for hematologic malignancies [2, 3, 4, 5, 6, 7, 8]. These agents have shown great efficacy in heavily pretreated populations [2, 3, 4, 5, 6, 7, 9].

The expanding use of BsAbs has prompted increasing attention to their toxicity profiles. Cytokine release syndrome (CRS) and neurotoxicity are well‐characterized complications and the focus of current management guidelines [10]. However, cardiovascular adverse events (CVAEs) are potentially significant but have not been thoroughly investigated. Early clinical trials were not powered to detect cardiovascular signals, and the reported incidence of CVAEs has varied considerably across studies [11].

Prior FAERS analyses have examined subsets of BsAbs grouped by indication or approval date [12, 13], precluding direct comparison across disease categories and target antigens. Whether cardiovascular risk varies by agent, target antigen, or underlying malignancy is unclear. In addition, the relationship between CVAEs and CRS or infection is incompletely defined; early data suggest most CVAEs occur independently of documented CRS, unlike CAR‐T therapy, where approximately two‐thirds overlap [11, 14, 15].

Herein, we performed a comprehensive disproportionality analysis of all FDA‐approved BsAbs for hematologic malignancies using the FDA Adverse Event Reporting System (FAERS) to characterize cardiovascular safety and establish a reference for the cardiovascular toxicity of these drugs.

## 2 Methods

### 2.1 Data Source

We conducted a retrospective pharmacovigilance study using FAERS, a publicly available database of spontaneous adverse event reports. FAERS data from December 2014 to September 2025 were included. Duplicate reports were identified and removed based on case identifiers, patient demographics, event dates, and reported drugs. The full non‐BsAb FAERS population served as the primary comparator group, consistent with the conventional pharmacovigilance approach [16, 17]. Active‐comparator restricted sensitivity analyses were additionally performed using FDA‐approved, guideline‐based hematologic malignancy therapies as comparators [18].

### 2.2 Study Population

Reports were classified as BsAb‐related if they listed blinatumomab, mosunetuzumab, epcoritamab, glofitamab, teclistamab, elranatamab, or talquetamab as the primary suspected drug. Generic and brand names were standardized to the active ingredient. Reports were excluded if age or sex was missing. Cardiovascular outcomes were identified using the Medical Dictionary for Regulatory Activities (MedDRA) Preferred Terms [19]. For outcomes with established Standardized MedDRA Queries (SMQs), the corresponding SMQ was used; for outcomes without an established SMQ, investigator‐defined Preferred Term groupings were used. Cardiovascular events were analyzed as both a composite (CVAE) and independently (separate cardiac and vascular categories). The complete list of MedDRA Preferred Terms for each subcategory is provided in Table S1.

### 2.3 Statistical Analysis

We performed a disproportionality analysis comparing BsAb‐exposed reports to all non‐BsAb‐related adverse event reports in FAERS (*N* = 7 289 316). Effect sizes were reported as adjusted reporting odds ratios (aRORs) derived from multivariable logistic regression models adjusted for age, sex, cardiovascular comorbidity, disease indication, and concomitant cardiotoxic medications. Age was modeled using restricted cubic splines with three knots when sufficient data were available; otherwise, linear age terms were used. Concomitantly listed cardiotoxic medications are listed in Table S2.

Analyses were conducted at three levels: (1) BsAbs overall, (2) by disease (B‐ALL, lymphoma, and MM), and (3) by individual drug. All models adjusted for disease indication. Within‐class head‐to‐head comparisons were performed among lymphoma BsAbs (reference: mosunetuzumab) and MM BsAbs (reference: teclistamab).

Among BsAb‐exposed patients, we assessed whether specific CVAEs were associated with death using logistic regression. Fatal CVAEs were defined as reports with a cardiovascular event in which death was also a reported outcome. Case‐fatality proportions were defined as the proportion of reported events with death as a recorded outcome and are not equivalent to population‐level mortality rates, given that severe outcomes are preferentially reported in passive surveillance and the population at risk is not directly observable.

For reports with available timing data (49.8% of BsAb CVAE reports; *N* = 701 of 1408), we calculated median time‐to‐event with interquartile range. Wilcoxon rank‐sum tests compared timing between cardiovascular and non‐cardiovascular events.

The proportion of cardiovascular events occurring concurrently with CRS and infection was calculated for each outcome, defined as the presence of the respective event and the cardiovascular outcome of interest in the same report.

#### 2.3.1 Sensitivity Analyses

Active‐comparator restricted sensitivity analyses, Firth penalized logistic regression for sparse outcomes, and Benjamini–Hochberg false discovery rate adjustment are described in the Supporting Information. Complete active‐comparator drug lists and full sensitivity analysis results are provided in Tables S3–S12.

All analyses were performed using R version 4.4.3 [20]; *p* < 0.05 denoted statistical significance.

## 3 Results

### 3.1 Study Population

Overall, we identified 7647 adverse event reports involving BsAbs approved for hematological malignancies from the FAERS database (Table 1). Of these, 3890 (50.9%) listed blinatumomab as the primary suspected drug, followed by teclistamab (954; 12.5%), epcoritamab (884; 11.6%), elranatamab (687; 9.0%), glofitamab (570; 7.5%), mosunetuzumab (351; 4.6%), and talquetamab (311; 4.1%). Based on the approved indication of the primary suspected drug, 3890 reports (50.9%) involved BsAbs approved for B‐ALL, 1805 (23.6%) for B‐cell lymphoma, and 1952 (25.5%) for multiple myeloma. Baseline characteristics of comparator reports are summarized in Table S3.

**TABLE 1:** Baseline characteristics of included FAERS reports.

		B‐ALL	Lymphoma			Multiple myeloma		
Characteristic	Overall	Blinatumomab	Mosunetuzumab	Epcoritamab	Glofitamab	Teclistamab	Elranatamab	Talquetamab
Total reports, *n*	7647	3890	351	884	570	954	687	311
Age, median (IQR), years	60 (36–71)	38 (19–60)	68 (59.5–76)	72 (63–79)	65 (54–73.75)	68 (61–75)	70 (61–76)	67 (60–74)
Sex, *n* (%)								
Male	4239 (55.4)	2120 (54.5)	207 (59)	516 (58.4)	357 (62.6)	490 (51.4)	369 (53.7)	180 (57.9)
Female	3408 (44.6)	1770 (45.5)	144 (41)	368 (41.6)	213 (37.4)	464 (48.6)	318 (46.3)	131 (42.1)
Unknown	0 (0)	0 (0)	0 (0)	0 (0)	0 (0)	0 (0)	0 (0)	0 (0)
Reporter, *n* (%)								
Healthcare professional	5778 (75.6)	2847 (73.2)	337 (96)	752 (85.1)	372 (65.3)	726 (76.1)	547 (79.6)	197 (63.3)
Consumer	622 (8.1)	226 (5.8)	7 (2)	40 (4.5)	169 (29.6)	65 (6.8)	54 (7.9)	61 (19.6)
Disease category, *n* (%)								
B‐ALL	3163 (41.4)	3158 (81.2)	3 (0.9)	1 (0.1)	1 (0.2)	0 (0)	0 (0)	0 (0)
Multiple myeloma	1650 (21.6)	3 (0.1)	0 (0)	0 (0)	0 (0)	863 (90.5)	523 (76.1)	261 (83.9)
DLBCL	1182 (15.5)	28 (0.7)	31 (8.8)	710 (80.3)	413 (72.5)	0 (0)	0 (0)	0 (0)
Follicular lymphoma	169 (2.2)	0 (0)	102 (29.1)	51 (5.8)	16 (2.8)	0 (0)	0 (0)	0 (0)
Other B‐cell lymphoma	49 (0.6)	14 (0.4)	6 (1.7)	6 (0.7)	23 (4)	0 (0)	0 (0)	0 (0)
Lymphoma NOS	30 (0.4)	3 (0.1)	0 (0)	8 (0.9)	19 (3.3)	0 (0)	0 (0)	0 (0)
Unknown/not documented	1404 (18.4)	684 (17.6)	209 (59.5)	108 (12.2)	98 (17.2)	91 (9.5)	164 (23.9)	50 (16.1)
Concomitant medications, *n* (%)								
BTK inhibitor	17 (0.2)	1 (0)	5 (1.4)	5 (0.6)	6 (1.1)	0 (0)	0 (0)	0 (0)
Anthracycline	296 (3.9)	180 (4.6)	40 (11.4)	25 (2.8)	49 (8.6)	1 (0.1)	1 (0.1)	0 (0)
Cyclophosphamide	371 (4.9)	239 (6.1)	41 (11.7)	30 (3.4)	52 (9.1)	5 (0.5)	2 (0.3)	2 (0.6)
CAR‐T therapy	27 (0.4)	16 (0.4)	0 (0)	4 (0.5)	5 (0.9)	1 (0.1)	0 (0)	1 (0.3)
IMiD	193 (2.5)	10 (0.3)	51 (14.5)	27 (3.1)	12 (2.1)	44 (4.6)	22 (3.2)	27 (8.7)
Proteasome inhibitor	46 (0.6)	24 (0.6)	0 (0)	0 (0)	1 (0.2)	9 (0.9)	8 (1.2)	4 (1.3)
Anti‐CD38 antibody	209 (2.7)	2 (0.1)	0 (0)	0 (0)	0 (0)	134 (14)	48 (7)	25 (8)
Cardiovascular comorbidity, *n* (%)	478 (6.3)	56 (1.4)	78 (22.2)	41 (4.6)	76 (13.3)	107 (11.2)	90 (13.1)	30 (9.6)

Abbreviations: B‐ALL, B‐cell acute lymphoblastic leukemia; BsAb, bispecific antibody; BTK, Bruton's tyrosine kinase; CAR‐T, chimeric antigen receptor T‐cell; DLBCL, diffuse large B‐cell lymphoma; IMiD, immunomodulatory drug.

**FIGURE 1:**
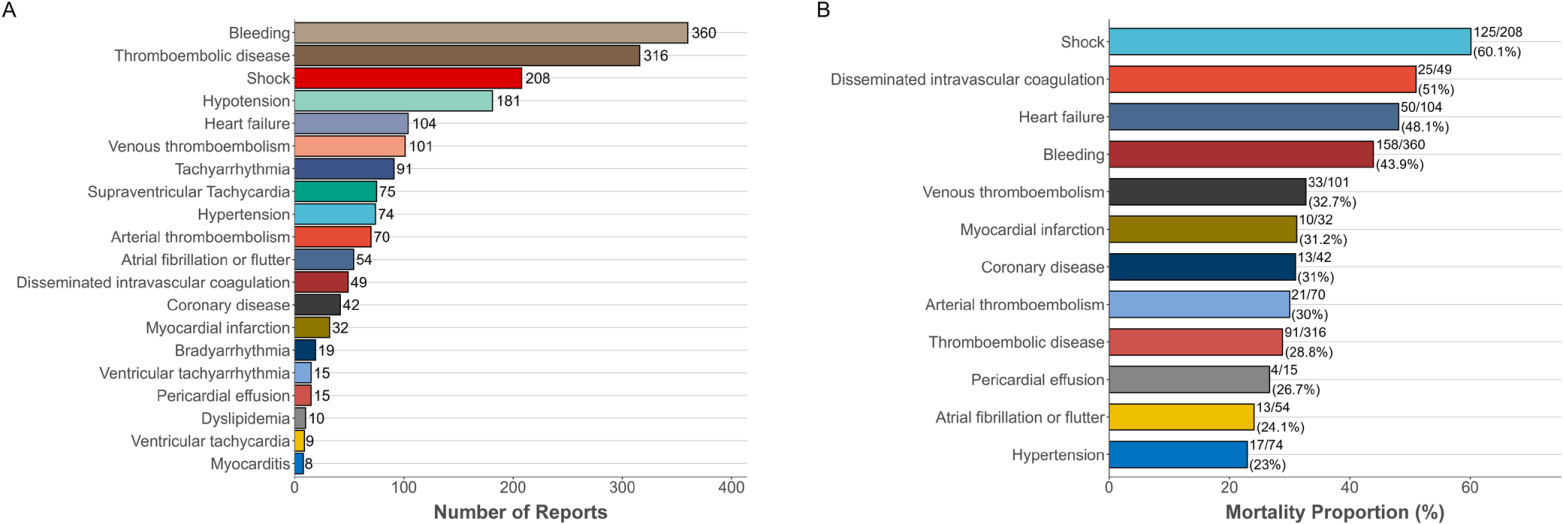
(A) Cardiovascular adverse events reported with bispecific antibodies. (B) Case fatality rates of cardiovascular events reported in bispecific antibody‐treated patients. [Color figure can be viewed at wileyonlinelibrary.com]

CVAEs were reported in 1408 cases (18.4%), with 447 (5.8%) fatal. CRS was reported in 1508 cases (19.7%). Vascular events (1046 reports; 13.7%) were more frequently reported than cardiac events (637 reports; 8.3%) (Figure 1A).

### 3.2 Overall

In multivariable logistic regression models, BsAbs as a class were associated with increased reporting of disseminated intravascular coagulation (DIC) (aROR: 4.35 [3.18–5.96]; *n* = 49), hypotension (aROR: 1.61 [1.38–1.89]; *n* = 181), and fatal CVAEs (aROR: 1.63 [1.47–1.80]; *n* = 447) (Table 2). BsAbs were not associated with increased reporting of overall CVAE (aROR: 0.68 [0.64–0.72]) or cardiac adverse events specifically (aROR: 0.63 [0.58–0.68]). These findings were consistent in a sensitivity analysis comparing BsAbs against pooled FDA‐approved hematologic malignancy therapies (Table S4).

**TABLE 2:** Cardiotoxicity signals for B‐ALL/lymphoma BsABs.

Outcome	B‐ALL		Lymphoma					
	Blinatumomab (*N*)	Blinatumomab (aROR)	Mosunetuzumab (*N*)	Mosunetuzumab (aROR)	Epcoritamab (*N*)	Epcoritamab (aROR)	Glofitamab (*N*)	Glofitamab (aROR)
CVAE	762	0.68 [0.62–0.74]	58	0.54 [0.41–0.72]	149	0.62 [0.52–0.74]	102	0.68 [0.55–0.85]
Fatal CVAE	200	1.22 [1.05–1.42]^a^	9	0.58 [0.3–1.12]	71	2.06 [1.61–2.64]^a^	40	1.82 [1.31–2.52]^a^
Cardiac adverse events	307	0.62 [0.55–0.70]	39	0.73 [0.52–1.01]	75	0.58 [0.45–0.73]	47	0.57 [0.42–0.77]
Heart failure	48	0.85 [0.63–1.15]	5	0.69 [0.29–1.68]	14	0.96 [0.57–1.64]	6	0.64 [0.28–1.43]
Myocarditis	2	0.77 [0.19–3.19]	—	—	—	—	1	2.46 [0.34–17.72]
Coronary disease	17	0.35 [0.21–0.57]	3	0.54 [0.17–1.69]	4	0.37 [0.14–0.98]	5	0.68 [0.28–1.66]
Myocardial infarction	12	0.37 [0.21–0.67]	2	0.5 [0.13–2.02]	3	0.39 [0.13–1.23]	5	0.99 [0.41–2.41]
Supraventricular tachycardia	19	0.67 [0.42–1.07]	10	2.21 [1.17–4.15]^a^	15	1.03 [0.62–1.73]	8	0.98 [0.49–1.98]
Atrial fibrillation or flutter	10	0.47 [0.25–0.89]	9	2.25 [1.15–4.38]^a^	11	0.83 [0.46–1.51]	5	0.71 [0.29–1.73]
Tachyarrhythmia	32	0.88 [0.61–1.27]	12	2.14 [1.2–3.82]^a^	16	0.93 [0.57–1.53]	8	0.79 [0.39–1.59]
Ventricular tachyarrhythmia	11	1.32 [0.7–2.47]	1	0.99 [0.14–7.07]	3	1.24 [0.4–3.90]	—	—
QT prolongation	7	0.96 [0.44–2.09]	—	—	—	—	—	—
Bradyarrhythmia	12	0.97 [0.54–1.75]	—	—	1	0.26 [0.04–1.82]	—	—
Pericardial effusion	11	0.59 [0.32–1.08]	—	—	1	0.71 [0.1–5.04]	1	0.91 [0.13–6.52]
Pericarditis	3	0.36 [0.11–1.16]	1	3.14 [0.44–22.47]	1	1.78 [0.25–12.82]	—	—
Vascular adverse events	603	0.71 [0.64–0.77]	33	0.44 [0.31–0.63]	102	0.64 [0.52–0.78]	70	0.69 [0.53–0.88]
Thromboembolic disease	224	0.79 [0.69–0.92]	11	0.59 [0.32–1.08]	24	0.6 [0.4–0.90]	12	0.45 [0.25–0.80]
Arterial thromboembolism	37	0.52 [0.37–0.73]	6	0.92 [0.41–2.07]	6	0.46 [0.21–1.03]	6	0.71 [0.32–1.59]
Venous thromboembolism	80	0.83 [0.65–1.04]	2	0.42 [0.1–1.69]	6	0.58 [0.26–1.31]	2	0.25 [0.06–1.02]
Bleeding	234	0.94 [0.82–1.09]	6	0.24 [0.11–0.53]	28	0.49 [0.33–0.71]	21	0.59 [0.38–0.92]
Cerebrovascular disease	4	0.9 [0.32–2.51]	—	—	—	—	—	—
Shock	84	0.69 [0.55–0.87]	6	0.68 [0.3–1.52]	26	1.3 [0.88–1.93]	18	1.25 [0.78–2.01]
Hypotension	97	1.46 [1.18–1.81]^a^	6	1.16 [0.52–2.61]	25	2.02 [1.35–3.03]^a^	10	1.27 [0.68–2.38]
Disseminated intravascular coagulation	38	4.41 [3.05–6.37]^a^	—	—	3	4.5 [1.43–14.20]^a^	—	—
Hypertension	47	0.47 [0.35–0.63]	—	—	6	0.45 [0.2–1.00]	3	0.34 [0.11–1.06]
Dyslipidemia	7	0.12 [0.06–0.25]	—	—	—	—	1	0.68 [0.1–4.89]

Abbreviation: aROR, adjusted reporting odds ratio.

^a^ Statistically significant increased reporting (*p* < 0.05).

**FIGURE 2:**
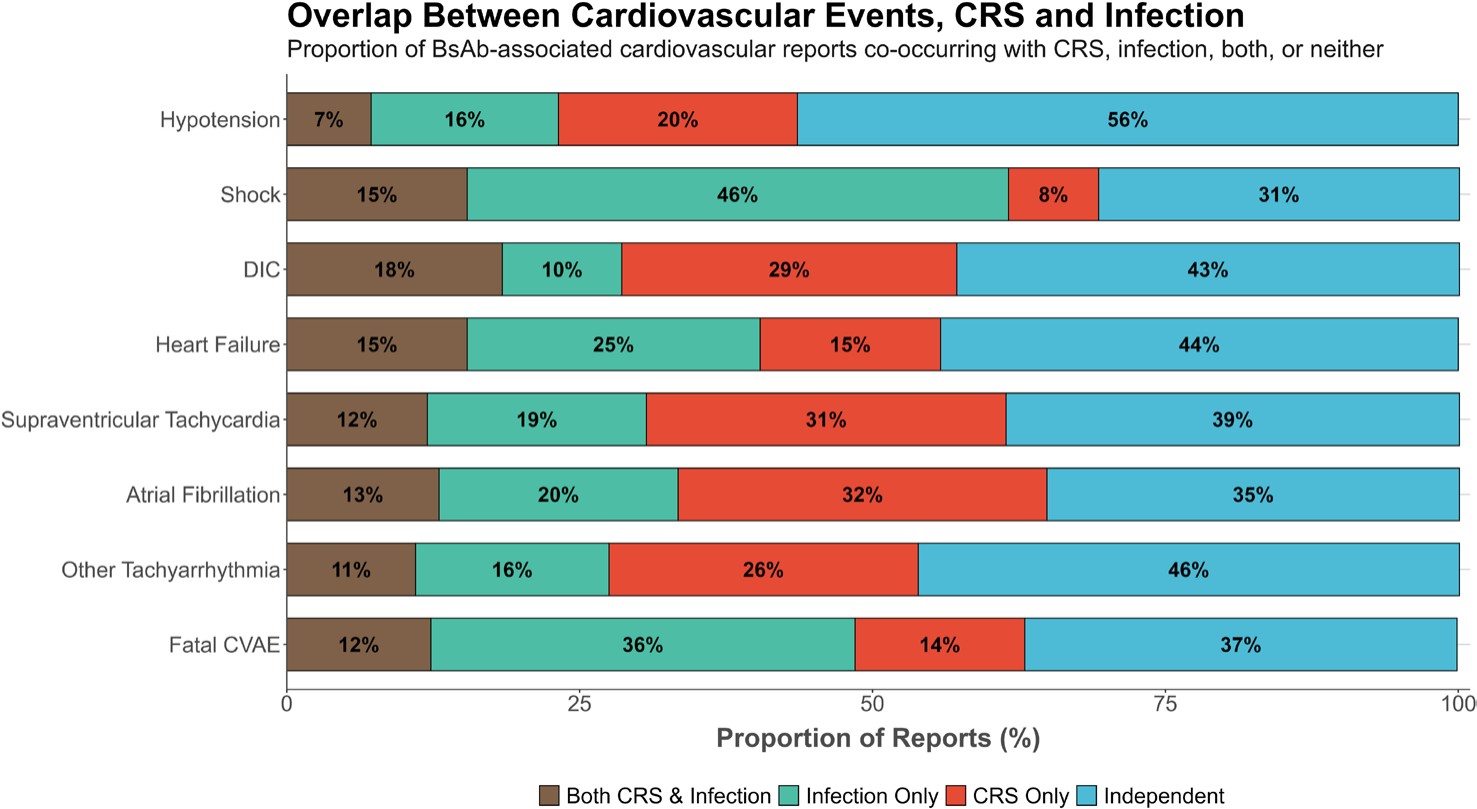
Overlap between CVAEs, CRS, and infection. [Color figure can be viewed at wileyonlinelibrary.com]

### 3.3 Drug‐Level Analyses

#### 3.3.1 BsAbs for B‐ALL

After adjustment for disease indication, blinatumomab was associated with increased reporting of DIC (aROR: 4.41 [3.05–6.37]; *n* = 38) and hypotension (aROR: 1.46 [1.18–1.81]; *n* = 97). Fatal CVAE reporting was also elevated (aROR: 1.22 [1.05–1.42]; *n* = 200) (Table 2). In the active‐comparator sensitivity analysis against B‐ALL‐directed therapies, all of the aforementioned signals retained statistical significance (Table S5).

#### 3.3.2 BsAbs for Non‐Hodgkin Lymphoma

Among the lymphoma‐specific BsAbs, cardiovascular reporting profiles varied (Table 2). Mosunetuzumab showed a consistent arrhythmogenic signal not observed with other BsAbs, with increased reporting of supraventricular tachycardia (aROR: 2.21 [1.17–4.15]; *n* = 10), atrial fibrillation or flutter (aROR: 2.25 [1.15–4.38]; *n* = 9), and tachyarrhythmia (aROR: 2.14 [1.20–3.82]; *n* = 12). These signals did not persist against a B‐NHL active comparator (Table S6).

Epcoritamab showed increased reporting of hypotension (aROR: 2.02 [1.35–3.03]; *n* = 25) and fatal CVAEs (aROR: 2.06 [1.61–2.64]; *n* = 71). Both signals persisted against the B‐NHL active comparator (hypotension aROR: 1.73 [1.15–2.61]; fatal CVAE aROR: 1.84 [1.43–2.36]) (Table S6).

Glofitamab showed no individual cardiovascular signals, though fatal CVAE reporting was significantly elevated (aROR: 1.82 [1.31–2.52]; *n* = 40), which also survived the active comparator analysis (aROR: 1.47 [1.06–2.04]) (Table S6).

In within‐class head‐to‐head comparisons using mosunetuzumab as the reference, no significant differences were identified between epcoritamab, glofitamab, and mosunetuzumab for any cardiovascular endpoint.

#### 3.3.3 BsAbs for Multiple Myeloma

MM‐targeting BsAbs as a class were associated with increased reporting of DIC (aROR: 5.21 [2.50–10.84]; *n* = 8), myocarditis (aROR: 3.68 [1.49–9.10]; *n* = 5), shock (aROR: 2.09 [1.65–2.66]; *n* = 74), hypotension (aROR: 1.81 [1.33–2.46]; *n* = 43), and fatal CVAEs (aROR: 2.04 [1.69–2.45]; *n* = 127) (Table 3). In the active‐comparator sensitivity analysis against MM‐directed therapies, all five class‐level signals persisted (Table S7).

**TABLE 3:** Cardiotoxicity signals for multiple myeloma BsABs.

Outcome	BCMA‐CD3				GPRC5D‐CD3	
	Teclistamab (*N*)	Teclistamab (aROR)	Elranatamab (*N*)	Elranatamab (aROR)	Talquetamab (*N*)	Talquetamab (aROR)
CVAE	170	0.55 [0.47–0.65]	110	0.48 [0.39–0.58]	57	0.59 [0.44–0.79]
Fatal CVAE	68	2.26 [1.76–2.91]^a^	44	1.9 [1.39–2.59]^a^	15	1.52 [0.9–2.57]
Cardiac adverse events	86	0.55 [0.44–0.68]	53	0.46 [0.35–0.61]	30	0.61 [0.42–0.89]
Heart failure	16	1.1 [0.67–1.81]	14	1.3 [0.76–2.22]	1	0.22 [0.03–1.56]
Myocarditis	4	5.95 [2.16–16.43]^a^	1	1.86 [0.26–13.33]	—	—
Coronary disease	9	0.55 [0.29–1.07]	3	0.25 [0.08–0.76]	1	0.18 [0.03–1.3]
Myocardial infarction	7	0.58 [0.27–1.22]	3	0.33 [0.11–1.03]	—	—
Supraventricular tachycardia	11	0.99 [0.54–1.8]	10	1.3 [0.7–2.44]	2	0.59 [0.15–2.38]
Atrial fibrillation or flutter	8	0.82 [0.4–1.64]	9	1.34 [0.69–2.59]	2	0.67 [0.17–2.71]
Tachyarrhythmia	11	0.81 [0.45–1.48]	10	1.06 [0.57–1.99]	2	0.48 [0.12–1.94]
Ventricular tachyarrhythmia	—	—	—	—	—	—
QT prolongation	1	0.93 [0.13–6.69]	—	—	—	—
Bradyarrhythmia	4	1.19 [0.44–3.2]	2	0.73 [0.18–2.93]	—	—
Pericardial effusion	2	1.23 [0.3–4.98]	—	—	—	—
Pericarditis	—	—	1	3.41 [0.47–24.54]	—	—
Vascular adverse events	117	0.55 [0.46–0.67]	74	0.47 [0.37–0.59]	47	0.73 [0.53–0.99]
Thromboembolic disease	25	0.43 [0.29–0.64]	12	0.28 [0.16–0.5]	8	0.42 [0.21–0.85]
Arterial thromboembolism	8	0.42 [0.21–0.84]	5	0.35 [0.15–0.85]	2	0.32 [0.08–1.28]
Venous thromboembolism	6	0.43 [0.19–0.97]	3	0.32 [0.1–0.99]	2	0.43 [0.11–1.72]
Bleeding	36	0.71 [0.51–0.99]	20	0.48 [0.31–0.75]	15	0.92 [0.55–1.55]
Cerebrovascular disease	—	—	—	—	1	40.01 [5.27–303.53]^a^
Shock	40	2.32 [1.68–3.2]^a^	29	2.27 [1.56–3.31]^a^	5	0.87 [0.36–2.11]
Hypotension	14	1.19 [0.7–2.03]	15	1.7 [1.02–2.85]^a^	14	3.79 [2.2–6.51]^a^
Disseminated intravascular coagulation	3	4.07 [1.27–13.01]^a^	3	5.47 [1.73–17.35]^a^	2	7.67 [1.86–31.64]^a^
Hypertension	8	0.4 [0.2–0.8]	9	0.62 [0.32–1.19]	1	0.16 [0.02–1.07]
Dyslipidemia	—	—	1	0.5 [0.07–3.55]	1	1.15 [0.16–8.25]

Abbreviation: aROR, adjusted reporting odds ratio.

^a^ Statistically significant increased reporting (*p* < 0.05).

Teclistamab showed increased reporting of myocarditis (aROR: 5.95 [2.16–16.43]; *n* = 4) and shock (aROR: 2.32 [1.68–3.20]; *n* = 40). The myocarditis signal persisted against the MM active comparator (aROR: 7.05 [2.25–17.19]). An association with fatal CVAEs was also seen (aROR: 2.26 [1.76–2.91]; *n* = 68).

Elranatamab showed increased reporting of shock (aROR: 2.27 [1.56–3.31]; *n* = 29), which persisted against the active comparator (aROR: 2.14 [1.46–3.13]). The signal for fatal CVAEs was also elevated (aROR: 1.90 [1.39–2.59]; *n* = 44). Talquetamab showed the highest hypotension signal among all BsAbs (aROR: 3.79 [2.20–6.51]; *n* = 14), which also persisted against the MM active comparator (aROR: 3.27 [1.90–5.64]) (Table S7).

In within‐class head‐to‐head comparisons using teclistamab as the reference, talquetamab was associated with significantly higher odds of reporting hypotension (aOR: 3.02 [1.40–6.51]; *p* = 0.004) but significantly lower odds of shock (aOR: 0.37 [0.13–0.86]; *p* = 0.037). No significant differences were observed between elranatamab and teclistamab for any cardiovascular outcome.

### 3.4 Time to Onset of BsAb‐Related CVAEs

CVAEs occurred sooner following BsAb therapy compared with non‐CVAEs (median time to onset: 7 vs. 15 days; *p* < 0.001). DIC and hypotension events occurred at a median time of 1 day and 2 days, respectively, following BsAb initiation. Heart failure occurred at a median of 4 days (IQR: 1–16), with 57.1% of events within 7 days. Shock had a later median onset of 10 days (IQR: 1–47), with 44.8% within 7 days. Fatal CVAEs occurred at a median of 10.5 days (IQR: 2–36), with 43.4% occurring within the first week. For reference, CRS had a median onset of 2 days (IQR: 1–8), with 72.2% occurring within 7 days. Time to CVAE onset differed significantly across individual drugs (Kruskal–Wallis *p* < 0.001; Table S8).

### 3.5 Case Fatality Proportions Associated With BsAb‐Related CVAEs

CVAEs were associated with a significantly higher proportion of mortality compared with non‐CVAEs (31.7% vs. 18.7%; *p* < 0.001). The CVAEs with the highest mortality proportions were shock (60.1%), DIC (51.0%), heart failure (48.1%), and bleeding (43.9%) (Figure 1B). Conversely, the mortality proportions associated with CRS were lower (24.3%).

Drug‐specific fatality proportions varied. Teclistamab‐associated myocarditis carried a 75% fatality rate (three of four cases). Shock fatality was highest with glofitamab (72.2%) and teclistamab (67.5%). Among lymphoma BsAbs, epcoritamab showed the highest overall CVAE case fatality (47.7%), while mosunetuzumab showed the lowest despite its arrhythmia signal.

Within the BsAb cohort, specific cardiovascular events were independently associated with death. Reporting of vascular events (aOR: 1.97 [1.70–2.29]) and cardiac events (aOR: 1.50 [1.25–1.81]) was associated with increased mortality. Shock showed the strongest association with mortality (aOR: 5.09 [3.80–6.80]), followed by DIC (aOR: 4.15 [2.34–7.35]), bleeding (aOR: 3.45 [2.75–4.34]), and heart failure (aOR: 2.93 [1.97–4.36]). This observation remained significant when adjusting for individual drug class (Table S9).

### 3.6 Co‐Occurrence of CRS and Infection

We examined co‐occurrence of CVAEs with CRS and infection within the same report. At the class level, the majority of hypotension reports (56.4%) occurred in the absence of both CRS and infection (CRS overlap: 27.6%; infection overlap: 23.2%). Heart failure reports occurred without concurrent CRS or infection in 44.2% of cases (CRS overlap: 30.8%; infection overlap: 40.4%). DIC reports occurred independently of both in 42.9% of cases (CRS overlap: 46.9%; infection overlap: 28.6%). Shock had the highest rate of co‐occurrence, with 61.5% of reports co‐occurring with infection and 23.1% with CRS; 30.8% of shock reports occurred in the absence of both. Among arrhythmia events, the majority occurred independently of both CRS and infection: SVT (42.7% CRS overlap; 39.7% independent), atrial fibrillation/flutter (44.4% CRS overlap; 36.5% independent), and tachyarrhythmia (37.4% CRS overlap; 47.2% independent) (Figure 2; Table S10).

Among fatal CVAEs, 26.8% co‐occurred with CRS. Drug‐specific CRS overlap patterns varied significantly across agents for hypotension (*p* = 0.049), tachyarrhythmia (*p* = 0.007), and fatal CVAEs (*p* < 0.001), but not for heart failure (*p* = 0.55), DIC (*p* = 0.22), SVT (*p* = 0.087), or atrial fibrillation (*p* = 0.11).

Epcoritamab showed the highest CRS‐cardiovascular overlap, with 56.0% of hypotension reports and 63.4% of fatal CVAEs occurring with concurrent CRS. Teclistamab‐associated myocarditis showed 75% CRS overlap (three of four cases). Among mosunetuzumab‐associated arrhythmias, the majority occurred independently of both CRS and infection (SVT: 70%; atrial fibrillation: 66.7%; tachyarrhythmia: 75%), supporting that this arrhythmogenic signal is not solely attributable to CRS‐mediated hemodynamic disturbance. In contrast, blinatumomab showed lower CRS overlap.

## 4 Discussion

To our knowledge, this is the largest cross‐agent pharmacovigilance analysis to systematically characterize cardiovascular safety signals across all FDA‐approved BsAbs for hematologic malignancies. Our analysis demonstrates that cardiovascular safety signals associated with BsAbs vary by agent, molecular target, and underlying malignancy. DIC was the most prominent class‐level cardiovascular signal, persisting against both pooled and disease‐specific active comparators. Notably, a significant proportion of events occurred independently of CRS or infection. Three important drug‐specific safety signals were seen. First, blinatumomab was associated with DIC after adjustment for disease indication. Second, mosunetuzumab demonstrated a consistent arrhythmogenic profile not observed with other CD20‐directed agents. These arrhythmia signals did not persist against a B‐NHL active comparator containing BTK inhibitors with established arrhythmogenic side effects [21], though results suggest that mosunetuzumab‐associated arrhythmia risk is comparable to, rather than absent relative to, other NHL‐directed therapies. Third, teclistamab was associated with increased reporting of myocarditis and shock.

These observations extend our prior analysis [13] and are supported by prospective data from 37 clinical trials (3758 patients) reporting tachycardia (10.2%), arrhythmias (12.6%), and hypotension (13.3%) as the most frequent CVAEs [22].

In our prior analysis, blinatumomab was primarily associated with DIC and hypotension [13]; the current study identifies DIC as the predominant signal with a larger sample and longer follow‐up. In addition, the observation that a large proportion of blinatumomab‐associated DIC events occurred in the absence of CRS or infection suggests that at least a subset may occur through CRS‐independent mechanisms [11].

The arrhythmogenic signal with mosunetuzumab is particularly notable, as it was not seen with epcoritamab or glofitamab despite their shared CD20‐CD3 target antigen, though within‐class head‐to‐head comparisons were not statistically significant, likely reflecting limited power for rare events. Budde et al. reported CRS in 44% of mosunetuzumab‐treated patients, including hypotension (38%) and tachycardia (28%) [6]. However, the signal identified in our analysis encompasses clinically significant arrhythmias such as atrial fibrillation. In addition, overlap between CRS and arrhythmia events was modest, supporting the relative independence of these events from CRS‐mediated hemodynamic perturbations.

Among MM‐directed BsAbs, the myocarditis signal observed with teclistamab is consistent with our earlier analysis [13] and independently supported by Shan et al., who reported positive signal strength for noninfectious myocarditis, cardiomyopathy, and cardiac failure in a FAERS‐based study of myeloma BsAbs [12, 13]. This signal strengthened in the active‐comparator sensitivity analysis against MM‐directed therapies, including carfilzomib and other known cardiotoxic agents, supporting a BsAb‐specific mechanism. Notably, no cases of myocarditis were identified on chart review in a clinical cohort of 567 TCE‐treated patients, and the underlying mechanisms remain undefined [23]. Consequently, given its rarity but clinical significance, this signal warrants validation in large‐scale studies.

The relationship between CVAEs and CRS has been central to understanding the cardiovascular toxicity of T‐cell engaging therapies. In our prior analysis, most CVAEs did not overlap with documented CRS [13]. The present study incorporates both CRS and infection co‐occurrence. Notably, the majority of hypotension and heart failure reports occurred in the absence of both CRS and infection, supporting the interpretation that these may represent distinct cardiovascular signals. In contrast, shock demonstrated the greatest co‐occurrence with infection, suggesting that a considerable proportion of these cases may be sepsis‐related. These patterns contrast with CAR T‐cell therapy, where 68.3% of cardiovascular events overlapped with CRS, suggesting that BsAb‐associated cardiovascular toxicity may arise more frequently through CRS‐independent mechanisms [15]. Conversely, in a cohort of 567 patients receiving BsAb therapy, development of Grade ≥ 2 CRS and/or ICANS was independently associated with a more than twofold increase in cardiovascular event risk when modeled as a time‐dependent covariate [23]. This discrepancy likely reflects methodological differences where time‐dependent modeling captures the downstream consequences of immune activation over subsequent days, whereas co‐occurrence in spontaneous reports captures only events documented simultaneously; together, these findings suggest that while severe CRS elevates subsequent cardiovascular risk, a substantial proportion of CVAEs are reported independent of CRS.

Fatal CVAE reporting was elevated across all agents except mosunetuzumab and talquetamab, and case fatality proportions for shock, DIC, and heart failure exceeded those observed with CRS and neurotoxicity. Current NCCN recommendations for lymphocyte engager‐related toxicities focus primarily on CRS grading, tocilizumab use, and corticosteroid management [10]; our findings suggest that cardiovascular‐specific monitoring, particularly during the early treatment period when most hemodynamic events occur, is warranted.

This study has several limitations. First, FAERS is a spontaneous reporting database subject to reporting bias, underreporting, an inability to establish causality, and lack of granular clinical data, including baseline cardiac function, biomarkers (e.g., troponin or NT‐proBNP), and serial imaging. Second, disproportionality analyses evaluate reporting patterns rather than true incidence; thus, elevated reporting odds ratios may reflect awareness or reporting practices rather than actual differences in risk, and findings should be interpreted as signals warranting prospective evaluation. Third, the use of the full non‐BsAb FAERS database as the primary comparator pools heterogeneous patient populations with differing baseline cardiovascular risk; while we addressed this through regression adjustment and disease‐specific active‐comparator sensitivity analyses, residual confounding cannot be excluded. Fourth, we could not fully account for the effects of traditional cardiovascular therapies (e.g., statins) on cardiac event development. Fifth, as with all pharmacovigilance analyses, some signals were based on small event counts and should be interpreted accordingly (Firth's penalized regression was used to address potential sparse‐data bias for outcomes with fewer than 10 events), though the detection of rare but clinically significant events is a strength of this methodology; however, it does not imply causality.

## 5 Conclusion

In conclusion, our findings provide the most comprehensive characterization to date of BsAb‐associated cardiotoxicity, addressing a critical gap given the limited prospective cardiovascular safety data for this class. Together with emerging evidence, these results underscore the need for systematic, prospective cardiovascular surveillance as BsAbs are increasingly used in earlier lines of therapy and in combination regimens [24, 25, 26]. Future studies should evaluate whether biomarkers, analogous to those proposed in CAR T‐cell therapy [11, 27, 28], can identify patients at highest risk; whether standardized cardiovascular monitoring strategies extending beyond current NCCN guidelines can improve outcomes; and whether elucidation of agent‐specific mechanisms can inform risk stratification in clinical practice.

## Funding

The authors have nothing to report.

## Ethics Statement

The authors have nothing to report.

## Consent

The authors have nothing to report.

## Conflicts of Interest

Daniel Addison is supported by NIH grants R01CA301579, R01HL168045, and R01HL170038 and by CPRIT grant RR250048. Dae Hyun Lee is supported by AHA CDA 24CDA1271837. The other authors declare no conflicts of interest.

## Supporting information


**Table S1:** MedDRA terms.
**Table S2:** Concomitant cardiotoxic medications and active comparator drug list.
**Table S3:** Baseline characteristics of comparator reports.
**Table S4:** BsAB versus pooled hematological malignancy therapies.
**Table S5:** Blinatumomab versus B‐ALL active comparator.
**Table S6:** Lymphoma BsAbs versus active comparator.
**Table S7:** Myeloma BsAbs versus active comparator.
**Table S8:** Time to cardiovascular vs. non‐cardiovascular events by individual drug.
**Table S9:** Mortality association with cardiovascular events.
**Table S10:** Co‐occurrence of CRS and infection with cardiovascular events by individual drug.
**Table S11:** Benjamini‐Hochberg FDR‐adjusted *p* values.
**Table S12:** Firth penalized logistic regression for sparse signals (*n* < 10).

## Data Availability

The data used in this analysis are publicly available from the FDA Adverse Event Reporting System (FAERS) at https://www.fda.gov/drugs/fdas‐adverse‐event‐reporting‐system‐faers/fda‐adverse‐event‐reporting‐system‐faers‐latest‐quarterly‐data‐files. The code for the statistical analysis is available from the first author upon request.
