# Supplementary material for: Cardiovascular Toxicity Associated With Bispecific Antibodies in Hematological Malignancies: A Comprehensive Pharmacovigilance Analysis
Source: Am J Hematol. 2026 Jul 24;101(10):2546–56. doi: 10.1002/ajh.70449 (PMC13540327; doi:10.1002/ajh.70449)
Supplement: Supplementary file 1 [file AJH-101-2546-s001.docx]

Supplementary Tables

**Contents**

[Supplementary Methods: Sensitivity Analyses	2](#_Toc234602294)

[S1. MedDRA Terms	3](#_Toc234602295)

[S2. Concomitant Cardiotoxic Medications and Active Comparator Drug List	7](#_Toc234602296)

[S3. Baseline Characteristics of Comparator Reports	7](#_Toc234602297)

[S4. BsAB versus Pooled Hematological Malignancy Therapies	8](#_Toc234602298)

[S5. Blinatumomab versus B-ALL Active Comparator	11](#_Toc234602299)

[S6. Lymphoma BsAbs versus Active Comparator	12](#_Toc234602300)

[S7. Myeloma BsAbs versus Active Comparator	15](#_Toc234602301)

[S8. Time to Cardiovascular vs Non-Cardiovascular Events by Individual Drug	19](#_Toc234602302)

[S9. Mortality Association with Cardiovascular Events	20](#_Toc234602303)

[S10. Co-Occurrence of CRS and Infection with Cardiovascular Events by Individual Drug	21](#_Toc234602304)

[S11. Benjamini-Hochberg FDR-Adjusted P-values.	23](#_Toc234602305)

[S12. Firth Penalized Logistic Regression for Sparse Signals (n<10).	25](#_Toc234602306)

## Supplementary Methods: Sensitivity Analyses

Active-comparator restricted disproportionality analyses were performed at two levels. First, BsAb-exposed reports were compared against reports listing any FDA-approved hematologic malignancy therapy as the primary suspected drug. Second, we performed disease-specific analyses, where blinatumomab was compared against B-ALL therapies; lymphoma-directed BsAbs against B-NHL therapies; and MM-directed BsAbs against MM therapies, with drug lists derived from current NCCN guidelines (Table S2). To address multiplicity, Benjamini–Hochberg false discovery rate adjustment was applied to primary-analysis p-values stratified by analytic family (class-level, disease-level, drug-level) (Table S11). To address potential sparse data, Firth penalized logistic regression was applied to drug-outcome combinations with fewer than 10 events in the BsAb arm (Table S12)..

## S1. MedDRA Terms

| **Adverse event** | **Terms used for its identification** |
| --- | --- |
| Arterial thromboembolism | Embolic and thrombotic events, arterial (SMQ) |
| Atrial fibrillation or flutter | "Atrial fibrillation", "Atrial flutter" |
| Bleeding | Haemorrhages (SMQ) |
| Bradyarrhythmia | Bradyarrhythmias (incl conduction defects and disorders of sinus node function) (SMQ) |
| Cardiac adverse events | All preferred terms corresponding to the System Organ Class "Cardiac Disorders" |
| Cerebrovascular disease | Central nervous system vascular disorders (SMQ) |
| Coronary disease | Ischaemic heart disease (SMQ) |
| Cytokine release syndrome | "Cytokine release syndrome", "Cytokine storm" |
| Disseminated intravascular coagulation | "Disseminated intravascular coagulation" |
| Dyslipidemia | Dyslipidaemia (SMQ) |
| Endocarditis | "Endocarditis", "Abiotrophia defectiva endocarditis", "Endocarditis candida", "Endocarditis enterococcal", "Endocarditis haemophilus", "Endocarditis histoplasma", "Endocarditis pseudomonal", "Endocarditis Q fever", "Endocarditis staphylococcal", "Endocarditis viral", "Fungal endocarditis", "Lupus endocarditis", "Prosthetic valve endocarditis", "Septic endocarditis", "Streptococcal endocarditis", "Subacute endocarditis" |
| GBS | Guillain-Barre syndrome (SMQ) |
| Gait disturbance | "Ataxia", "Cerebellar ataxia", "Cerebral ataxia", "Hemiataxia", "Vestibular ataxia", "Autoimmune cerebellar ataxia", "Ataxia with vitamin E deficiency", "Decreased gait velocity", "Gait apraxia", "Gait deviation", "Gait disturbance", "Gait inability", "Gait spastic", "Parkinsonian gait", "Propulsive gait", "Tandem gait test abnormal" |
| Headache | "Headache", "Cervicogenic headache", "Cluster headache", "Cold-stimulus headache", "Drug withdrawal headache", "Exertional headache", "External compression headache", "Hypnic headache", "Ictal epileptic headache", "Medication overuse headache", "Menstrual headache", "New daily persistent headache", "Postictal headache", "Post-traumatic headache", "Primary cough headache", "Primary headache associated with sexual activity", "Procedural headache", "Sinus headache", "Tension headache", "Thunderclap headache", "Vascular headache", "Migraine", "Migraine postdrome", "Migraine with aura", "Migraine without aura", "Hemiplegic migraine", "Basilar migraine", "Migrainous infarction", "Ophthalmic migraine", "Ophthalmoplegic migraine", "Status migrainosus", "Retinal migraine", "Vestibular migraine", "Chronic paroxysmal hemicrania", "Occipital neuralgia", "SUNA syndrome", "SUNCT syndrome" |
| Heart failure | Cardiac failure (SMQ) |
| Hypertension | Hypertension (SMQ) |
| Hypotension | "Hypotension", "CT hypotension complex", "Dialysis hypotension", "Diastolic hypotension", "Neonatal hypotension", "Orthostatic hypotension", "Post procedural hypotension", "Procedural hypotension" |
| Infection | All preferred terms corresponding to the System Organ Class "Infections and Infestations" |
| Myocardial infarction | Myocardial infarction (SMQ) |
| Myocarditis | "Myocarditis", "Autoimmune myocarditis", "Chronic myocarditis", "Coxsackie myocarditis", "Cytomegalovirus myocarditis", "Enterovirus myocarditis", "Eosinophilic myocarditis", "Giant cell myocarditis", "Hypersensitivity myocarditis", "Immune-mediated myocarditis", "Lupus myocarditis", "Malarial myocarditis", "Myocarditis bacterial", "Myocarditis helminthic", "Myocarditis infectious", "Myocarditis meningococcal", "Myocarditis mycotic", "Myocarditis post infection", "Myocarditis septic", "Myocarditis syphilitic", "Myocarditis toxoplasmal", "Radiation myocarditis", "Viral myocarditis" |
| Neurotoxicity | All preferred terms corresponding to the System Organ Class "Nervous system disorders" |
| Pericardial effusion | "Pericardial effusion", "Cardiac tamponade" |
| Pericarditis | "Pericarditis", "Pericarditis adhesive", "Pericarditis constrictive", "Pleuropericarditis", "Myopericarditis", "Autoimmune pericarditis", "Immune-mediated pericarditis" |
| QT Prolongation | "Electrocardiogram QT interval abnormal", "Electrocardiogram QT prolonged", "Long QT syndrome" |
| Seizure | Convulsions (SMQ) |
| Shock | Shock (SMQ) |
| Speech disturbance | "Speech disorder", "Poverty of speech", "Pedantic speech", "Disorganised speech", "Slow speech", "Repetitive speech", "Speech disorder developmental", "Speech latency", "Dysarthria", "Aphasia", "Acquired epileptic aphasia", "Primary progressive aphasia", "Transient aphasia" |
| Sudden death | "Sudden death", "Sudden cardiac death" |
| Supraventricular Tachycardia | Supraventricular tachyarrhythmias (SMQ) |
| Tachyarrhythmia | Tachyarrhythmias (incl supraventricular and ventricular tachyarrhythmias) (SMQ) |
| Thromboembolic disease | Embolic and thrombotic events (SMQ) |
| Tremor | "Tremor", "Action tremor", "Dystonic tremor", "Essential tremor", "Holmes tremor", "Intention tremor", "Orthostatic tremor", "Parkinsonian rest tremor", "Postural tremor", "Psychogenic tremor", "Resting tremor", "Tremor neonatal" |
| Valvular disease | "Aortic valve disease mixed", "Aortic valve incompetence", "Aortic valve sclerosis", "Aortic valve stenosis", "Bicuspid aortic valve", "Congenital aortic valve incompetence", "Congenital aortic valve stenosis", "Subvalvular aortic stenosis", "Supravalvular aortic stenosis", "Heyde's syndrome", "Williams syndrome", "Aortic valve calcification", "Aortic valve prolapse", "Aortic valve disease", "Aortic valve atresia", "Degenerative aortic valve disease", "Aortic valve thickening", "Aortic annulus rupture", "Unicuspid aortic valve", "Congenital mitral valve incompetence", "Congenital mitral valve stenosis", "Mitral valve disease mixed", "Mitral valve incompetence", "Mitral valve prolapse", "Mitral valve stenosis", "Mitral valve calcification", "Mitral valve sclerosis", "Mitral valve disease", "Parachute mitral valve", "Mitral valve atresia", "Mitral perforation", "Mitral face", "Degenerative mitral valve disease", "Systolic anterior motion of mitral valve", "Myxomatous mitral valve degeneration", "Ischaemic mitral regurgitation", "Mitral valve thickening", "Mitral valve dysplasia", "Pulmonary valve incompetence", "Pulmonary valve stenosis", "Pulmonary valve stenosis congenital", "Congenital pulmonary valve atresia", "Pulmonary valve calcification", "Pulmonary valve sclerosis", "Congenital pulmonary valve disorder", "Pulmonary valve disease", "Bicuspid pulmonary valve", "Pulmonary valve thickening", "Congenital tricuspid valve stenosis", "Tricuspid valve incompetence", "Tricuspid valve stenosis", "Congenital tricuspid valve atresia", "Tricuspid valve calcification", "Tricuspid valve sclerosis", "Tricuspid valve disease", "Tricuspid valve prolapse", "Congenital tricuspid valve incompetence", "Degenerative tricuspid valve disease", "Tricuspid valve thickening", "Straddling tricuspid valve", "Tricuspid valve disease mixed", "Tricuspid valve thrombosis", "Cardiac valve replacement complication", "Cardiac valve vegetation", "Heart valve calcification", "Cardiac valve sclerosis", "Cardiac valve disease", "Heart valve stenosis", "Congenital heart valve disorder", "Cardiac valve abscess", "Shone complex", "Heart valve incompetence", "Cardiac valve rupture", "Carcinoid heart disease", "Congenital heart valve incompetence", "Cardiac valve discolouration", "Cardiac valve thickening", "Degenerative multivalvular disease", "Lambl's excrescences", "Cardiac valve fatty infiltration", "Prosthetic cardiac valve regurgitation" |
| Vascular adverse events | All preferred terms corresponding to the System Organ Class "Vascular disorders" |
| Vasculitis | Vasculitis (SMQ) |
| Venous thromboembolism | Embolic and thrombotic events, venous (SMQ) |
| Ventricular extrasystole | "Ventricular parasystole", "Ventricular extrasystoles" |
| Ventricular fibrillation | "Ventricular fibrillation" |
| Ventricular tachyarrhythmia | Ventricular tachyarrhythmias (SMQ) |
| Ventricular tachycardia | "Ventricular tachycardia", "Torsade de pointes" |
| All of the above terms refer to terms within the "Preferred Terms" level of the MedDRA heirarchy unless noted otherwise. "Standardized Medical Queries" (SMQs) and "System Organ Classes" refer to higher-level concepts in the MedDRA heirarchy which multiple Preferred Terms corresponding to the adverse event are placed under. | |
| SMQ: Standardized Medical Query | |

## S2. Concomitant Cardiotoxic Medications and Active Comparator Drug List

| **Bruton tyrosine kinase inhibitors** | ibrutinib, acalabrutinib, zanubrutinib |
| --- | --- |
| **Anthracyclines** | doxorubicin, daunorubicin, idarubicin, epirubicin |
| **CAR-T therapy** | tisagenlecleucel, axicabtagene ciloleucel, lisocabtagene maraleucel, brexucabtagene autoleucel, idecabtagene vicleucel, ciltacabtagene autoleucel |
| **immunomodulatory drugs** | lenalidomide, pomalidomide, thalidomide |
| **proteasome inhibitors** | bortezomib, carfilzomib, ixazomib |
| **anti-CD38 antibodies** | daratumumab, isatuximab |
| **B-ALL Active comparator (vs blinatumomab)** | vincristine, daunorubicin, doxorubicin, idarubicin, mitoxantrone, cyclophosphamide, ifosfamide, methotrexate, cytarabine, etoposide, fludarabine, mercaptopurine, pegaspargase, asparaginase, clofarabine, imatinib, dasatinib, ponatinib, nilotinib, bosutinib, asciminib, inotuzumab ozogamicin, rituximab, venetoclax, revumenib, tisagenlecleucel, brexucabtagene autoleucel, obecabtagene autoleucel. |
| **B-NHL Active comparator (vs mosunetuzumab, epcoritamab, glofitamab)** | rituximab, obinutuzumab, cyclophosphamide, doxorubicin, vincristine, bendamustine, etoposide, ifosfamide, gemcitabine, oxaliplatin, cisplatin, carboplatin, cytarabine, ibrutinib, zanubrutinib, pirtobrutinib, polatuzumab vedotin, loncastuximab tesirine, brentuximab vedotin, tafasitamab, venetoclax, lenalidomide, tazemetostat, axicabtagene ciloleucel, tisagenlecleucel, lisocabtagene maraleucel. |
| **MM Active comparator (vs teclistamab, elranatamab, talquetamab)** | bortezomib, carfilzomib, ixazomib, lenalidomide, pomalidomide, thalidomide, daratumumab, isatuximab, elotuzumab, belantamab mafodotin, venetoclax, cyclophosphamide, melphalan, bendamustine, doxorubicin, idecabtagene vicleucel, ciltacabtagene autoleucel. |

## S3. Baseline Characteristics of Comparator Reports

| **Variable** | **BsAb cohort** | **Full FAERS comparator** | **Pooled Heme Drugs** | **B-ALL comparator** | **B-NHL comparator** | **MM comparator** |
| --- | --- | --- | --- | --- | --- | --- |
| N | 7,647 | 7,289,316 | 535,410 | 163,010 | 353,469 | 289,524 |
| Median age (IQR) | 60 (36–71) | 59 (42–70) | 66 (55–74) | 57 (37–68) | 67 (57–75) | 70 (61–77) |
| Female n (%) | 3,408 (44.6%) | 4,338,916 (59.5%) | 271,088 (50.6%) | 92,068 (56.5%) | 171,576 (48.5%) | 141,362 (48.8%) |
| Death n (%) | 1,613 (24.0%) | 516,072 (11.4%) | 51,455 (12.5%) | 17,821 (12.5%) | 35,320 (13.0%) | 25,262 (12.9%) |
| CV comorbidity n (%) | 478 (6.3%) | 803,502 (11.0%) | 26,868 (5.0%) | 9,556 (5.9%) | 18,737 (5.3%) | 13,137 (4.5%) |
| CRS n (%) | 1,508 (19.7%) | 9,064 (0.1%) | 6,854 (1.3%) | 2,692 (1.7%) | 5,065 (1.4%) | 1,447 (0.5%) |
| Infection n (%) | 2,002 (26.2%) | 1,075,539 (14.8%) | 123,696 (23.1%) | 43,869 (26.9%) | 78,624 (22.2%) | 65,876 (22.8%) |
| Anthracycline n (%) | 296 (3.9%) | 71,496 (1.0%) | 40,046 (7.5%) | 31,971 (19.6%) | 34,249 (9.7%) | 19,064 (6.6%) |
| BTKi n (%) | 17 (0.2%) | 9,453 (0.1%) | 6,989 (1.3%) | 1,635 (1.0%) | 6,647 (1.9%) | 1,428 (0.5%) |
| Cyclophos n (%) | 371 (4.9%) | 85,326 (1.2%) | 45,449 (8.5%) | 32,291 (19.8%) | 34,831 (9.9%) | 23,045 (8.0%) |
| CAR-T n (%) | 27 (0.4%) | 11,072 (0.2%) | 10,423 (1.9%) | 3,731 (2.3%) | 7,607 (2.2%) | 1,883 (0.7%) |

## S4. BsAB versus Pooled Hematological Malignancy Therapies

| **Drug** | **Outcome** | **N (BsAb)** | **Events** | **N (comparator)** | **aROR (95% CI)** | **P-value** |
| --- | --- | --- | --- | --- | --- | --- |
| All BsAbs (overall) | Fatal CVAE | 7,647 | 447 | 535,410 | 1.60 (1.45 to 1.78) | <0.001 |
| B-ALL BsAbs (class) | Fatal CVAE | 3,890 | 200 | 535,410 | 1.28 (1.10 to 1.50) | 0.002 |
| Lymphoma BsAbs (class) | Fatal CVAE | 1,805 | 120 | 535,410 | 1.56 (1.28 to 1.88) | <0.001 |
| MM BsAbs (class) | Fatal CVAE | 1,952 | 127 | 535,410 | 1.78 (1.47 to 2.14) | <0.001 |
| Blinatumomab | Fatal CVAE | 3,890 | 200 | 535,410 | 1.28 (1.10 to 1.50) | 0.002 |
| Epcoritamab | Fatal CVAE | 884 | 71 | 535,410 | 2.03 (1.58 to 2.61) | <0.001 |
| Glofitamab | Fatal CVAE | 570 | 40 | 535,410 | 1.58 (1.14 to 2.19) | 0.006 |
| Teclistamab | Fatal CVAE | 954 | 68 | 535,410 | 2.01 (1.56 to 2.58) | <0.001 |
| Elranatamab | Fatal CVAE | 687 | 44 | 535,410 | 1.67 (1.22 to 2.27) | 0.001 |
| Talquetamab | Fatal CVAE | 311 | 15 | 535,410 | 1.40 (0.83 to 2.36) | 0.208 |
| All BsAbs (overall) | Hypotension | 7,647 | 181 | 535,410 | 1.42 (1.21 to 1.66) | <0.001 |
| B-ALL BsAbs (class) | Hypotension | 3,890 | 97 | 535,410 | 1.26 (1.01 to 1.57) | 0.041 |
| Lymphoma BsAbs (class) | Hypotension | 1,805 | 41 | 535,410 | 1.49 (1.08 to 2.04) | 0.014 |
| MM BsAbs (class) | Hypotension | 1,952 | 43 | 535,410 | 1.50 (1.10 to 2.05) | 0.010 |
| Blinatumomab | Hypotension | 3,890 | 97 | 535,410 | 1.26 (1.01 to 1.57) | 0.041 |
| Mosunetuzumab | Hypotension | 351 | 6 | 535,410 | 1.11 (0.46 to 2.23) | 0.789 |
| Epcoritamab | Hypotension | 884 | 25 | 535,410 | 1.95 (1.30 to 2.93) | 0.001 |
| Glofitamab | Hypotension | 570 | 10 | 535,410 | 1.12 (0.60 to 2.11) | 0.721 |
| Teclistamab | Hypotension | 954 | 14 | 535,410 | 1.02 (0.60 to 1.73) | 0.951 |
| Elranatamab | Hypotension | 687 | 15 | 535,410 | 1.44 (0.86 to 2.41) | 0.165 |
| Talquetamab | Hypotension | 311 | 14 | 535,410 | 3.19 (1.86 to 5.48) | <0.001 |
| All BsAbs (overall) | Shock | 7,647 | 208 | 535,410 | 1.10 (0.95 to 1.28) | 0.189 |
| Lymphoma BsAbs (class) | Shock | 1,805 | 50 | 535,410 | 1.03 (0.78 to 1.38) | 0.816 |
| MM BsAbs (class) | Shock | 1,952 | 74 | 535,410 | 1.70 (1.33 to 2.16) | <0.001 |
| Epcoritamab | Shock | 884 | 26 | 535,410 | 1.24 (0.83 to 1.84) | 0.294 |
| Glofitamab | Shock | 570 | 18 | 535,410 | 1.07 (0.67 to 1.72) | 0.772 |
| Teclistamab | Shock | 954 | 40 | 535,410 | 1.90 (1.38 to 2.63) | <0.001 |
| Elranatamab | Shock | 687 | 29 | 535,410 | 1.86 (1.28 to 2.71) | 0.001 |
| All BsAbs (overall) | Disseminated intravascular coagulation | 7,647 | 49 | 535,410 | 3.54 (2.55 to 4.90) | <0.001 |
| B-ALL BsAbs (class) | Disseminated intravascular coagulation | 3,890 | 38 | 535,410 | 4.09 (2.76 to 6.06) | <0.001 |
| Lymphoma BsAbs (class) | Disseminated intravascular coagulation | 1,805 | 3 | 535,410 | 1.21 (0.34 to 3.03) | 0.734 |
| MM BsAbs (class) | Disseminated intravascular coagulation | 1,952 | 8 | 535,410 | 3.02 (1.36 to 5.87) | 0.009 |
| Blinatumomab | Disseminated intravascular coagulation | 3,890 | 38 | 535,410 | 4.09 (2.76 to 6.06) | <0.001 |
| Epcoritamab | Disseminated intravascular coagulation | 884 | 3 | 535,410 | 2.84 (0.78 to 7.22) | 0.101 |
| Teclistamab | Disseminated intravascular coagulation | 954 | 3 | 535,410 | 2.61 (0.71 to 6.72) | 0.130 |
| Elranatamab | Disseminated intravascular coagulation | 687 | 3 | 535,410 | 3.46 (0.95 to 8.80) | 0.057 |
| Talquetamab | Disseminated intravascular coagulation | 311 | 2 | 535,410 | 5.71 (1.18 to 16.69) | 0.034 |
| Epcoritamab | Heart failure | 884 | 14 | 535,410 | 1.05 (0.61 to 1.80) | 0.856 |
| Elranatamab | Heart failure | 687 | 14 | 535,410 | 1.14 (0.67 to 1.94) | 0.634 |
| All BsAbs (overall) | Myocarditis | 7,647 | 8 | 535,410 | 1.73 (0.79 to 3.28) | 0.154 |
| B-ALL BsAbs (class) | Myocarditis | 3,890 | 2 | 535,410 | 1.06 (0.21 to 3.25) | 0.928 |
| MM BsAbs (class) | Myocarditis | 1,952 | 5 | 535,410 | 3.40 (1.25 to 7.45) | 0.020 |
| Blinatumomab | Myocarditis | 3,890 | 2 | 535,410 | 1.06 (0.21 to 3.25) | 0.928 |
| Glofitamab | Myocarditis | 570 | 1 | 535,410 | 3.45 (0.39 to 12.79) | 0.212 |
| Teclistamab | Myocarditis | 954 | 4 | 535,410 | 5.87 (1.90 to 13.91) | 0.004 |
| Elranatamab | Myocarditis | 687 | 1 | 535,410 | 2.54 (0.29 to 9.29) | 0.325 |
| Glofitamab | Myocardial infarction | 570 | 5 | 535,410 | 1.26 (0.47 to 2.68) | 0.606 |
| Lymphoma BsAbs (class) | Supraventricular tachycardia | 1,805 | 33 | 535,410 | 1.02 (0.72 to 1.46) | 0.895 |
| Mosunetuzumab | Supraventricular tachycardia | 351 | 10 | 535,410 | 1.45 (0.77 to 2.73) | 0.255 |
| Mosunetuzumab | Atrial fibrillation or flutter | 351 | 9 | 535,410 | 1.57 (0.76 to 2.84) | 0.208 |
| Mosunetuzumab | Tachyarrhythmia | 351 | 12 | 535,410 | 1.51 (0.84 to 2.70) | 0.167 |
| All BsAbs (overall) | Bradyarrhythmia | 7,647 | 19 | 535,410 | 1.01 (0.63 to 1.62) | 0.954 |
| B-ALL BsAbs (class) | Bradyarrhythmia | 3,890 | 12 | 535,410 | 1.41 (0.75 to 2.64) | 0.282 |
| MM BsAbs (class) | Bradyarrhythmia | 1,952 | 6 | 535,410 | 1.34 (0.55 to 2.69) | 0.487 |
| Blinatumomab | Bradyarrhythmia | 3,890 | 12 | 535,410 | 1.41 (0.75 to 2.64) | 0.282 |
| Teclistamab | Bradyarrhythmia | 954 | 4 | 535,410 | 1.95 (0.65 to 4.43) | 0.208 |
| Elranatamab | Bradyarrhythmia | 687 | 2 | 535,410 | 1.38 (0.29 to 3.91) | 0.632 |
| All BsAbs (overall) | Cerebrovascular disease | 7,647 | 5 | 535,410 | 1.07 (0.39 to 2.45) | 0.880 |
| MM BsAbs (class) | Cerebrovascular disease | 1,952 | 1 | 535,410 | 4.25 (0.42 to 20.20) | 0.181 |
| Talquetamab | Cerebrovascular disease | 311 | 1 | 535,410 | 26.66 (2.67 to 125.03) | 0.011 |
| Mosunetuzumab | Pericarditis | 351 | 1 | 535,410 | 1.87 (0.21 to 6.78) | 0.491 |
| Epcoritamab | Pericarditis | 884 | 1 | 535,410 | 1.30 (0.15 to 4.85) | 0.764 |
| Elranatamab | Pericarditis | 687 | 1 | 535,410 | 2.05 (0.23 to 7.49) | 0.435 |
| All BsAbs (overall) | Endocarditis | 7,647 | 4 | 535,410 | 1.50 (0.49 to 3.52) | 0.438 |
| B-ALL BsAbs (class) | Endocarditis | 3,890 | 2 | 535,410 | 2.04 (0.39 to 6.86) | 0.349 |
| MM BsAbs (class) | Endocarditis | 1,952 | 2 | 535,410 | 1.98 (0.40 to 5.98) | 0.347 |
| Blinatumomab | Endocarditis | 3,890 | 2 | 535,410 | 2.04 (0.39 to 6.86) | 0.349 |
| Teclistamab | Endocarditis | 954 | 1 | 535,410 | 2.41 (0.27 to 9.26) | 0.357 |
| Talquetamab | Endocarditis | 311 | 1 | 535,410 | 8.13 (0.91 to 30.80) | 0.058 |
| Glofitamab | Pericardial effusion | 570 | 1 | 535,410 | 1.03 (0.12 to 3.72) | 0.976 |

## S5. Blinatumomab versus B-ALL Active Comparator

| **Outcome** | **N (blina)** | **Events** | **N (comparator)** | **aROR (95% CI)** | **P-value** |
| --- | --- | --- | --- | --- | --- |
| Composite CVAE | 3,890 | 762 | 163,010 | 0.79 (0.72 to 0.86) | <0.001 |
| Fatal CVAE | 3,890 | 200 | 163,010 | 1.34 (1.14 to 1.57) | <0.001 |
| Hypotension | 3,890 | 97 | 163,010 | 1.90 (1.51 to 2.39) | <0.001 |
| Shock | 3,890 | 84 | 163,010 | 0.74 (0.59 to 0.93) | 0.011 |
| Disseminated intravascular coagulation | 3,890 | 38 | 163,010 | 4.56 (3.04 to 6.84) | <0.001 |
| Heart failure | 3,890 | 48 | 163,010 | 1.07 (0.78 to 1.46) | 0.672 |
| Myocarditis | 3,890 | 2 | 163,010 | 1.40 (0.28 to 4.44) | 0.633 |
| Myocardial infarction | 3,890 | 12 | 163,010 | 0.48 (0.26 to 0.88) | 0.018 |
| Coronary disease | 3,890 | 17 | 163,010 | 0.42 (0.25 to 0.70) | <0.001 |
| Supraventricular tachycardia | 3,890 | 19 | 163,010 | 0.78 (0.48 to 1.29) | 0.337 |
| Atrial fibrillation or flutter | 3,890 | 10 | 163,010 | 0.69 (0.35 to 1.37) | 0.284 |
| Tachyarrhythmia | 3,890 | 32 | 163,010 | 1.11 (0.75 to 1.64) | 0.610 |
| Bradyarrhythmia | 3,890 | 12 | 163,010 | 1.63 (0.85 to 3.11) | 0.138 |
| Venous thromboembolism | 3,890 | 80 | 163,010 | 0.90 (0.70 to 1.15) | 0.391 |
| Arterial thromboembolism | 3,890 | 37 | 163,010 | 0.59 (0.42 to 0.85) | 0.004 |
| Thromboembolic disease | 3,890 | 224 | 163,010 | 0.89 (0.77 to 1.04) | 0.137 |
| Bleeding | 3,890 | 234 | 163,010 | 1.15 (0.99 to 1.34) | 0.062 |
| Cerebrovascular disease | 3,890 | 4 | 163,010 | 0.73 (0.23 to 1.78) | 0.513 |
| Pericarditis | 3,890 | 3 | 163,010 | 0.42 (0.11 to 1.10) | 0.081 |
| Endocarditis | 3,890 | 2 | 163,010 | 1.39 (0.27 to 4.67) | 0.653 |
| Pericardial effusion | 3,890 | 11 | 163,010 | 0.64 (0.34 to 1.21) | 0.173 |
| Cardiac adverse events | 3,890 | 307 | 163,010 | 0.76 (0.67 to 0.86) | <0.001 |
| Vascular adverse events | 3,890 | 603 | 163,010 | 0.81 (0.73 to 0.89) | <0.001 |

## S6. Lymphoma BsAbs versus Active Comparator

| **Drug** | **Outcome** | **N (BsAb)** | **Events** | **N (comparator)** | **aROR (95% CI)** | **P-value** |
| --- | --- | --- | --- | --- | --- | --- |
| Lymphoma BsAbs (class) | Composite CVAE | 1,805 | 309 | 353,469 | 0.61 (0.53 to 0.69) | <0.001 |
| Mosunetuzumab | Composite CVAE | 351 | 58 | 353,469 | 0.48 (0.36 to 0.64) | <0.001 |
| Epcoritamab | Composite CVAE | 884 | 149 | 353,469 | 0.64 (0.54 to 0.77) | <0.001 |
| Glofitamab | Composite CVAE | 570 | 102 | 353,469 | 0.65 (0.52 to 0.80) | <0.001 |
| Lymphoma BsAbs (class) | Fatal CVAE | 1,805 | 120 | 353,469 | 1.44 (1.19 to 1.75) | <0.001 |
| Mosunetuzumab | Fatal CVAE | 351 | 9 | 353,469 | 0.57 (0.28 to 1.02) | 0.061 |
| Epcoritamab | Fatal CVAE | 884 | 71 | 353,469 | 1.84 (1.43 to 2.36) | <0.001 |
| Glofitamab | Fatal CVAE | 570 | 40 | 353,469 | 1.47 (1.06 to 2.04) | 0.022 |
| Lymphoma BsAbs (class) | Hypotension | 1,805 | 41 | 353,469 | 1.32 (0.96 to 1.81) | 0.086 |
| Mosunetuzumab | Hypotension | 351 | 6 | 353,469 | 0.99 (0.41 to 1.98) | 0.982 |
| Epcoritamab | Hypotension | 884 | 25 | 353,469 | 1.73 (1.15 to 2.61) | 0.008 |
| Glofitamab | Hypotension | 570 | 10 | 353,469 | 1.01 (0.54 to 1.89) | 0.987 |
| Lymphoma BsAbs (class) | Shock | 1,805 | 50 | 353,469 | 0.91 (0.69 to 1.22) | 0.539 |
| Mosunetuzumab | Shock | 351 | 6 | 353,469 | 0.58 (0.24 to 1.17) | 0.139 |
| Epcoritamab | Shock | 884 | 26 | 353,469 | 1.07 (0.72 to 1.59) | 0.742 |
| Glofitamab | Shock | 570 | 18 | 353,469 | 0.95 (0.59 to 1.53) | 0.838 |
| Lymphoma BsAbs (class) | Disseminated intravascular coagulation | 1,805 | 3 | 353,469 | 1.11 (0.31 to 2.79) | 0.852 |
| Epcoritamab | Disseminated intravascular coagulation | 884 | 3 | 353,469 | 2.56 (0.70 to 6.53) | 0.135 |
| Lymphoma BsAbs (class) | Heart failure | 1,805 | 25 | 353,469 | 0.81 (0.54 to 1.21) | 0.307 |
| Mosunetuzumab | Heart failure | 351 | 5 | 353,469 | 0.72 (0.27 to 1.53) | 0.430 |
| Epcoritamab | Heart failure | 884 | 14 | 353,469 | 1.01 (0.59 to 1.72) | 0.982 |
| Glofitamab | Heart failure | 570 | 6 | 353,469 | 0.67 (0.27 to 1.33) | 0.273 |
| Lymphoma BsAbs (class) | Myocarditis | 1,805 | 1 | 353,469 | 0.86 (0.10 to 3.13) | 0.848 |
| Glofitamab | Myocarditis | 570 | 1 | 353,469 | 2.96 (0.33 to 10.98) | 0.265 |
| Lymphoma BsAbs (class) | Myocardial infarction | 1,805 | 10 | 353,469 | 0.67 (0.36 to 1.26) | 0.212 |
| Mosunetuzumab | Myocardial infarction | 351 | 2 | 353,469 | 0.64 (0.13 to 1.81) | 0.451 |
| Epcoritamab | Myocardial infarction | 884 | 3 | 353,469 | 0.53 (0.15 to 1.31) | 0.189 |
| Glofitamab | Myocardial infarction | 570 | 5 | 353,469 | 1.25 (0.47 to 2.66) | 0.615 |
| Lymphoma BsAbs (class) | Coronary disease | 1,805 | 12 | 353,469 | 0.55 (0.31 to 0.97) | 0.039 |
| Mosunetuzumab | Coronary disease | 351 | 3 | 353,469 | 0.62 (0.17 to 1.54) | 0.342 |
| Epcoritamab | Coronary disease | 884 | 4 | 353,469 | 0.46 (0.15 to 1.04) | 0.062 |
| Glofitamab | Coronary disease | 570 | 5 | 353,469 | 0.83 (0.31 to 1.76) | 0.666 |
| Lymphoma BsAbs (class) | Supraventricular tachycardia | 1,805 | 33 | 353,469 | 0.95 (0.67 to 1.36) | 0.785 |
| Mosunetuzumab | Supraventricular tachycardia | 351 | 10 | 353,469 | 1.31 (0.69 to 2.47) | 0.406 |
| Epcoritamab | Supraventricular tachycardia | 884 | 15 | 353,469 | 0.90 (0.53 to 1.52) | 0.694 |
| Glofitamab | Supraventricular tachycardia | 570 | 8 | 353,469 | 0.82 (0.38 to 1.52) | 0.553 |
| Lymphoma BsAbs (class) | Atrial fibrillation or flutter | 1,805 | 25 | 353,469 | 0.81 (0.54 to 1.21) | 0.302 |
| Mosunetuzumab | Atrial fibrillation or flutter | 351 | 9 | 353,469 | 1.43 (0.69 to 2.59) | 0.309 |
| Epcoritamab | Atrial fibrillation or flutter | 884 | 11 | 353,469 | 0.72 (0.39 to 1.33) | 0.294 |
| Glofitamab | Atrial fibrillation or flutter | 570 | 5 | 353,469 | 0.61 (0.23 to 1.29) | 0.213 |
| Lymphoma BsAbs (class) | Tachyarrhythmia | 1,805 | 36 | 353,469 | 0.90 (0.64 to 1.27) | 0.552 |
| Mosunetuzumab | Tachyarrhythmia | 351 | 12 | 353,469 | 1.37 (0.76 to 2.45) | 0.293 |
| Epcoritamab | Tachyarrhythmia | 884 | 16 | 353,469 | 0.85 (0.51 to 1.40) | 0.520 |
| Glofitamab | Tachyarrhythmia | 570 | 8 | 353,469 | 0.69 (0.32 to 1.29) | 0.267 |
| Lymphoma BsAbs (class) | Bradyarrhythmia | 1,805 | 1 | 353,469 | 0.28 (0.03 to 0.99) | 0.048 |
| Epcoritamab | Bradyarrhythmia | 884 | 1 | 353,469 | 0.68 (0.08 to 2.46) | 0.612 |
| Lymphoma BsAbs (class) | Venous thromboembolism | 1,805 | 10 | 353,469 | 0.27 (0.14 to 0.50) | <0.001 |
| Mosunetuzumab | Venous thromboembolism | 351 | 2 | 353,469 | 0.37 (0.08 to 1.03) | 0.058 |
| Epcoritamab | Venous thromboembolism | 884 | 6 | 353,469 | 0.36 (0.15 to 0.73) | 0.003 |
| Glofitamab | Venous thromboembolism | 570 | 2 | 353,469 | 0.19 (0.04 to 0.53) | <0.001 |
| Lymphoma BsAbs (class) | Arterial thromboembolism | 1,805 | 18 | 353,469 | 0.59 (0.37 to 0.95) | 0.031 |
| Mosunetuzumab | Arterial thromboembolism | 351 | 6 | 353,469 | 0.84 (0.34 to 1.68) | 0.649 |
| Epcoritamab | Arterial thromboembolism | 884 | 6 | 353,469 | 0.49 (0.20 to 0.99) | 0.047 |
| Glofitamab | Arterial thromboembolism | 570 | 6 | 353,469 | 0.70 (0.29 to 1.40) | 0.334 |
| Lymphoma BsAbs (class) | Thromboembolic disease | 1,805 | 47 | 353,469 | 0.46 (0.34 to 0.62) | <0.001 |
| Mosunetuzumab | Thromboembolic disease | 351 | 11 | 353,469 | 0.53 (0.29 to 0.96) | 0.038 |
| Epcoritamab | Thromboembolic disease | 884 | 24 | 353,469 | 0.50 (0.33 to 0.76) | 0.001 |
| Glofitamab | Thromboembolic disease | 570 | 12 | 353,469 | 0.36 (0.20 to 0.64) | <0.001 |
| Lymphoma BsAbs (class) | Bleeding | 1,805 | 55 | 353,469 | 0.54 (0.41 to 0.71) | <0.001 |
| Mosunetuzumab | Bleeding | 351 | 6 | 353,469 | 0.26 (0.11 to 0.53) | <0.001 |
| Epcoritamab | Bleeding | 884 | 28 | 353,469 | 0.62 (0.42 to 0.90) | 0.013 |
| Glofitamab | Bleeding | 570 | 21 | 353,469 | 0.67 (0.43 to 1.04) | 0.073 |
| Lymphoma BsAbs (class) | Pericarditis | 1,805 | 2 | 353,469 | 0.96 (0.20 to 2.78) | 0.943 |
| Mosunetuzumab | Pericarditis | 351 | 1 | 353,469 | 2.22 (0.25 to 8.07) | 0.393 |
| Epcoritamab | Pericarditis | 884 | 1 | 353,469 | 1.34 (0.15 to 5.07) | 0.735 |
| Lymphoma BsAbs (class) | Pericardial effusion | 1,805 | 2 | 353,469 | 0.62 (0.13 to 1.76) | 0.413 |
| Epcoritamab | Pericardial effusion | 884 | 1 | 353,469 | 0.89 (0.10 to 3.26) | 0.887 |
| Glofitamab | Pericardial effusion | 570 | 1 | 353,469 | 1.09 (0.12 to 3.95) | 0.921 |
| Lymphoma BsAbs (class) | Cardiac adverse events | 1,805 | 161 | 353,469 | 0.61 (0.52 to 0.72) | <0.001 |
| Mosunetuzumab | Cardiac adverse events | 351 | 39 | 353,469 | 0.65 (0.47 to 0.92) | 0.013 |
| Epcoritamab | Cardiac adverse events | 884 | 75 | 353,469 | 0.61 (0.48 to 0.78) | <0.001 |
| Glofitamab | Cardiac adverse events | 570 | 47 | 353,469 | 0.58 (0.43 to 0.79) | <0.001 |
| Lymphoma BsAbs (class) | Vascular adverse events | 1,805 | 205 | 353,469 | 0.59 (0.51 to 0.69) | <0.001 |
| Mosunetuzumab | Vascular adverse events | 351 | 33 | 353,469 | 0.40 (0.28 to 0.57) | <0.001 |
| Epcoritamab | Vascular adverse events | 884 | 102 | 353,469 | 0.66 (0.54 to 0.82) | <0.001 |
| Glofitamab | Vascular adverse events | 570 | 70 | 353,469 | 0.65 (0.50 to 0.84) | <0.001 |

## S7. Myeloma BsAbs versus Active Comparator

| **Drug** | **Outcome** | **N (BsAb)** | **Events** | **N (comparator)** | **aROR (95% CI)** | **P-value** |
| --- | --- | --- | --- | --- | --- | --- |
| MM BsAbs (class) | Composite CVAE | 1,952 | 337 | 289,524 | 0.60 (0.53 to 0.67) | <0.001 |
| Teclistamab | Composite CVAE | 954 | 170 | 289,524 | 0.62 (0.52 to 0.73) | <0.001 |
| Elranatamab | Composite CVAE | 687 | 110 | 289,524 | 0.54 (0.44 to 0.66) | <0.001 |
| Talquetamab | Composite CVAE | 311 | 57 | 289,524 | 0.67 (0.50 to 0.89) | 0.006 |
| MM BsAbs (class) | Fatal CVAE | 1,952 | 127 | 289,524 | 1.59 (1.31 to 1.92) | <0.001 |
| Teclistamab | Fatal CVAE | 954 | 68 | 289,524 | 1.80 (1.40 to 2.33) | <0.001 |
| Elranatamab | Fatal CVAE | 687 | 44 | 289,524 | 1.47 (1.08 to 2.01) | 0.015 |
| Talquetamab | Fatal CVAE | 311 | 15 | 289,524 | 1.26 (0.75 to 2.13) | 0.383 |
| MM BsAbs (class) | Hypotension | 1,952 | 43 | 289,524 | 1.52 (1.11 to 2.09) | 0.009 |
| Teclistamab | Hypotension | 954 | 14 | 289,524 | 1.02 (0.60 to 1.74) | 0.946 |
| Elranatamab | Hypotension | 687 | 15 | 289,524 | 1.47 (0.88 to 2.48) | 0.144 |
| Talquetamab | Hypotension | 311 | 14 | 289,524 | 3.27 (1.90 to 5.64) | <0.001 |
| MM BsAbs (class) | Shock | 1,952 | 74 | 289,524 | 1.93 (1.51 to 2.47) | <0.001 |
| Teclistamab | Shock | 954 | 40 | 289,524 | 2.14 (1.54 to 2.98) | <0.001 |
| Elranatamab | Shock | 687 | 29 | 289,524 | 2.14 (1.46 to 3.13) | <0.001 |
| Talquetamab | Shock | 311 | 5 | 289,524 | 0.95 (0.36 to 2.00) | 0.896 |
| MM BsAbs (class) | Disseminated intravascular coagulation | 1,952 | 8 | 289,524 | 2.56 (1.13 to 5.12) | 0.026 |
| Teclistamab | Disseminated intravascular coagulation | 954 | 3 | 289,524 | 2.23 (0.60 to 5.86) | 0.201 |
| Elranatamab | Disseminated intravascular coagulation | 687 | 3 | 289,524 | 2.90 (0.79 to 7.54) | 0.098 |
| Talquetamab | Disseminated intravascular coagulation | 311 | 2 | 289,524 | 4.91 (1.00 to 14.61) | 0.049 |
| MM BsAbs (class) | Heart failure | 1,952 | 31 | 289,524 | 0.86 (0.60 to 1.23) | 0.408 |
| Teclistamab | Heart failure | 954 | 16 | 289,524 | 0.91 (0.55 to 1.51) | 0.727 |
| Elranatamab | Heart failure | 687 | 14 | 289,524 | 1.05 (0.62 to 1.80) | 0.853 |
| Talquetamab | Heart failure | 311 | 1 | 289,524 | 0.27 (0.03 to 0.95) | 0.039 |
| MM BsAbs (class) | Myocarditis | 1,952 | 5 | 289,524 | 4.13 (1.49 to 9.34) | 0.009 |
| Teclistamab | Myocarditis | 954 | 4 | 289,524 | 7.05 (2.25 to 17.19) | 0.002 |
| Elranatamab | Myocarditis | 687 | 1 | 289,524 | 3.11 (0.35 to 11.65) | 0.247 |
| MM BsAbs (class) | Myocardial infarction | 1,952 | 10 | 289,524 | 0.48 (0.25 to 0.90) | 0.022 |
| Teclistamab | Myocardial infarction | 954 | 7 | 289,524 | 0.75 (0.33 to 1.45) | 0.421 |
| Elranatamab | Myocardial infarction | 687 | 3 | 289,524 | 0.46 (0.13 to 1.15) | 0.104 |
| MM BsAbs (class) | Coronary disease | 1,952 | 13 | 289,524 | 0.46 (0.26 to 0.80) | 0.006 |
| Teclistamab | Coronary disease | 954 | 9 | 289,524 | 0.70 (0.34 to 1.26) | 0.251 |
| Elranatamab | Coronary disease | 687 | 3 | 289,524 | 0.34 (0.10 to 0.84) | 0.016 |
| Talquetamab | Coronary disease | 311 | 1 | 289,524 | 0.35 (0.04 to 1.25) | 0.122 |
| MM BsAbs (class) | Supraventricular tachycardia | 1,952 | 23 | 289,524 | 0.75 (0.49 to 1.14) | 0.181 |
| Teclistamab | Supraventricular tachycardia | 954 | 11 | 289,524 | 0.72 (0.39 to 1.32) | 0.286 |
| Elranatamab | Supraventricular tachycardia | 687 | 10 | 289,524 | 0.92 (0.49 to 1.74) | 0.808 |
| Talquetamab | Supraventricular tachycardia | 311 | 2 | 289,524 | 0.56 (0.12 to 1.59) | 0.316 |
| MM BsAbs (class) | Atrial fibrillation or flutter | 1,952 | 19 | 289,524 | 0.71 (0.45 to 1.13) | 0.150 |
| Teclistamab | Atrial fibrillation or flutter | 954 | 8 | 289,524 | 0.65 (0.30 to 1.21) | 0.188 |
| Elranatamab | Atrial fibrillation or flutter | 687 | 9 | 289,524 | 1.03 (0.50 to 1.85) | 0.939 |
| Talquetamab | Atrial fibrillation or flutter | 311 | 2 | 289,524 | 0.65 (0.14 to 1.85) | 0.470 |
| MM BsAbs (class) | Tachyarrhythmia | 1,952 | 23 | 289,524 | 0.66 (0.44 to 1.01) | 0.055 |
| Teclistamab | Tachyarrhythmia | 954 | 11 | 289,524 | 0.64 (0.35 to 1.16) | 0.143 |
| Elranatamab | Tachyarrhythmia | 687 | 10 | 289,524 | 0.81 (0.43 to 1.53) | 0.522 |
| Talquetamab | Tachyarrhythmia | 311 | 2 | 289,524 | 0.49 (0.10 to 1.39) | 0.209 |
| MM BsAbs (class) | Bradyarrhythmia | 1,952 | 6 | 289,524 | 1.67 (0.68 to 3.40) | 0.239 |
| Teclistamab | Bradyarrhythmia | 954 | 4 | 289,524 | 2.42 (0.80 to 5.55) | 0.108 |
| Elranatamab | Bradyarrhythmia | 687 | 2 | 289,524 | 1.75 (0.36 to 5.00) | 0.423 |
| MM BsAbs (class) | Venous thromboembolism | 1,952 | 11 | 289,524 | 0.35 (0.19 to 0.64) | <0.001 |
| Teclistamab | Venous thromboembolism | 954 | 6 | 289,524 | 0.42 (0.17 to 0.84) | 0.012 |
| Elranatamab | Venous thromboembolism | 687 | 3 | 289,524 | 0.33 (0.09 to 0.80) | 0.011 |
| Talquetamab | Venous thromboembolism | 311 | 2 | 289,524 | 0.48 (0.10 to 1.35) | 0.187 |
| MM BsAbs (class) | Arterial thromboembolism | 1,952 | 15 | 289,524 | 0.45 (0.27 to 0.75) | 0.002 |
| Teclistamab | Arterial thromboembolism | 954 | 8 | 289,524 | 0.52 (0.24 to 0.96) | 0.036 |
| Elranatamab | Arterial thromboembolism | 687 | 5 | 289,524 | 0.45 (0.17 to 0.94) | 0.032 |
| Talquetamab | Arterial thromboembolism | 311 | 2 | 289,524 | 0.48 (0.10 to 1.36) | 0.192 |
| MM BsAbs (class) | Thromboembolic disease | 1,952 | 45 | 289,524 | 0.40 (0.30 to 0.54) | <0.001 |
| Teclistamab | Thromboembolic disease | 954 | 25 | 289,524 | 0.46 (0.31 to 0.68) | <0.001 |
| Elranatamab | Thromboembolic disease | 687 | 12 | 289,524 | 0.30 (0.17 to 0.54) | <0.001 |
| Talquetamab | Thromboembolic disease | 311 | 8 | 289,524 | 0.47 (0.22 to 0.87) | 0.014 |
| MM BsAbs (class) | Bleeding | 1,952 | 71 | 289,524 | 0.88 (0.69 to 1.12) | 0.298 |
| Teclistamab | Bleeding | 954 | 36 | 289,524 | 0.94 (0.67 to 1.32) | 0.727 |
| Elranatamab | Bleeding | 687 | 20 | 289,524 | 0.66 (0.42 to 1.04) | 0.072 |
| Talquetamab | Bleeding | 311 | 15 | 289,524 | 1.23 (0.73 to 2.08) | 0.434 |
| MM BsAbs (class) | Cerebrovascular disease | 1,952 | 1 | 289,524 | 11.45 (1.00 to 71.11) | 0.050 |
| Talquetamab | Cerebrovascular disease | 311 | 1 | 289,524 | 71.80 (6.57 to 399.74) | 0.003 |
| MM BsAbs (class) | Pericarditis | 1,952 | 1 | 289,524 | 1.86 (0.21 to 7.19) | 0.500 |
| Elranatamab | Pericarditis | 687 | 1 | 289,524 | 4.64 (0.52 to 17.83) | 0.139 |
| MM BsAbs (class) | Endocarditis | 1,952 | 2 | 289,524 | 2.77 (0.55 to 8.82) | 0.187 |
| Teclistamab | Endocarditis | 954 | 1 | 289,524 | 3.29 (0.36 to 13.27) | 0.235 |
| Talquetamab | Endocarditis | 311 | 1 | 289,524 | 11.51 (1.27 to 45.19) | 0.034 |
| MM BsAbs (class) | Pericardial effusion | 1,952 | 2 | 289,524 | 0.62 (0.13 to 1.80) | 0.422 |
| Teclistamab | Pericardial effusion | 954 | 2 | 289,524 | 1.28 (0.26 to 3.75) | 0.713 |
| MM BsAbs (class) | Cardiac adverse events | 1,952 | 169 | 289,524 | 0.52 (0.45 to 0.61) | <0.001 |
| Teclistamab | Cardiac adverse events | 954 | 86 | 289,524 | 0.54 (0.43 to 0.68) | <0.001 |
| Elranatamab | Cardiac adverse events | 687 | 53 | 289,524 | 0.45 (0.34 to 0.60) | <0.001 |
| Talquetamab | Cardiac adverse events | 311 | 30 | 289,524 | 0.62 (0.42 to 0.90) | 0.013 |
| MM BsAbs (class) | Vascular adverse events | 1,952 | 238 | 289,524 | 0.69 (0.60 to 0.79) | <0.001 |
| Teclistamab | Vascular adverse events | 954 | 117 | 289,524 | 0.70 (0.57 to 0.85) | <0.001 |
| Elranatamab | Vascular adverse events | 687 | 74 | 289,524 | 0.59 (0.46 to 0.76) | <0.001 |
| Talquetamab | Vascular adverse events | 311 | 47 | 289,524 | 0.91 (0.67 to 1.25) | 0.575 |

## S8. Time to Cardiovascular vs Non-Cardiovascular Events by Individual Drug

| **Drug** | **CVAE (N)** | **CVAE (median)** | **CVAE (IQR)** | **Non-CVAE (N)** | **Non-CVAE (median)** | **Non-CVAE**  **(IQR)** | **P-value** | |
| --- | --- | --- | --- | --- | --- | --- | --- | --- |
| Blinatumomab | 311 | 2 | 0-14 | 998 | 6.5 | 1-31.75 | <0.001 | |
| Mosunetuzumab | 43 | 44 | 7.5-81.5 | 251 | 27.0 | 7-115 | 0.9296 | |
| Epcoritamab | 83 | 9 | 3-20.5 | 339 | 15.0 | 6-42 | 0.0023 | |
| Glofitamab | 61 | 8 | 1-36 | 234 | 16.0 | 1-79 | 0.1326 | |
| Teclistamab | 101 | 32 | 2-95 | 469 | 43.0 | 6-164 | 0.0441 | |
| Elranatamab | 66 | 9 | 2-51.5 | 354 | 21.0 | 2-60 | 0.093 | |
| Talquetamab | 36 | 5 | 1-21.25 | 141 | 12.0 | 3-58 | 0.013 | |
| P-values from Wilcoxon rank-sum tests. IQR: interquartile range. | | | | | | | |  |

## S9. Mortality Association with Cardiovascular Events

| **Outcome** | **N_events** | **Base**  **OR (95% CI)** | **P (base)** | **Drug-Adjusted**  **OR (95% CI)** | **P (adj)** |
| --- | --- | --- | --- | --- | --- |
| CVAE | 1,335 | 1.93 (1.69-2.2) | <0.001 | 1.93 (1.69-2.21) | <0.001 |
| Cardiac adverse events | 600 | 1.49 (1.24-1.8) | <0.001 | 1.5 (1.25-1.81) | <0.001 |
| Vascular adverse events | 993 | 1.96 (1.69-2.27) | <0.001 | 1.97 (1.7-2.29) | <0.001 |
| Shock | 208 | 5.18 (3.88-6.92) | <0.001 | 5.09 (3.8-6.8) | <0.001 |
| DIC | 49 | 4.1 (2.32-7.27) | <0.001 | 4.15 (2.34-7.35) | <0.001 |
| Heart Failure | 104 | 3 (2.02-4.46) | <0.001 | 2.93 (1.97-4.36) | <0.001 |
| Bleeding | 336 | 3.41 (2.71-4.28) | <0.001 | 3.45 (2.75-4.34) | <0.001 |
| Base model adjusts for age (RCS), sex, disease category, and concomitant cardiotoxic medications. Drug-adjusted model additionally includes individual drug product. OR: odds ratio. | | | | | |

## S10. Co-Occurrence of CRS and Infection with Cardiovascular Events by Individual Drug

| **Drug** | **Outcome** | **N** | **CRS Only** | **Infection Only** | **Both** | **Independent** |
| --- | --- | --- | --- | --- | --- | --- |
| Blinatumomab | Hypotension | 97 | 10 (10.3%) | 18 (18.6%) | 5 (5.2%) | 64 (66%) |
| Blinatumomab | Shock | 84 | 6 (7.1%) | 33 (39.3%) | 10 (11.9%) | 35 (41.7%) |
| Blinatumomab | DIC | 38 | 11 (28.9%) | 3 (7.9%) | 5 (13.2%) | 19 (50%) |
| Blinatumomab | Heart Failure | 48 | 5 (10.4%) | 15 (31.2%) | 5 (10.4%) | 23 (47.9%) |
| Blinatumomab | Supraventricular Tachycardia | 19 | 3 (15.8%) | 5 (26.3%) | 1 (5.3%) | 10 (52.6%) |
| Blinatumomab | Atrial Fibrillation | 10 | 1 (10%) | 3 (30%) | 1 (10%) | 5 (50%) |
| Blinatumomab | Other Tachyarrhythmia | 32 | 4 (12.5%) | 6 (18.8%) | 2 (6.2%) | 20 (62.5%) |
| Blinatumomab | Fatal CVAE | 200 | 21 (10.5%) | 72 (36%) | 13 (6.5%) | 94 (47%) |
| Mosunetuzumab | Hypotension | 6 | 1 (16.7%) | 2 (33.3%) | 1 (16.7%) | 2 (33.3%) |
| Mosunetuzumab | Shock | 6 | 0 (0%) | 4 (66.7%) | 1 (16.7%) | 1 (16.7%) |
| Mosunetuzumab | Heart Failure | 5 | 1 (20%) | 1 (20%) | 0 (0%) | 3 (60%) |
| Mosunetuzumab | Supraventricular Tachycardia | 10 | 1 (10%) | 1 (10%) | 1 (10%) | 7 (70%) |
| Mosunetuzumab | Atrial Fibrillation | 9 | 1 (11.1%) | 1 (11.1%) | 1 (11.1%) | 6 (66.7%) |
| Mosunetuzumab | Other Tachyarrhythmia | 12 | 1 (8.3%) | 1 (8.3%) | 1 (8.3%) | 9 (75%) |
| Mosunetuzumab | Fatal CVAE | 9 | 0 (0%) | 7 (77.8%) | 0 (0%) | 2 (22.2%) |
| Epcoritamab | Hypotension | 25 | 8 (32%) | 3 (12%) | 6 (24%) | 8 (32%) |
| Epcoritamab | Shock | 26 | 3 (11.5%) | 12 (46.2%) | 7 (26.9%) | 4 (15.4%) |
| Epcoritamab | DIC | 3 | 1 (33.3%) | 1 (33.3%) | 1 (33.3%) | 0 (0%) |
| Epcoritamab | Heart Failure | 14 | 1 (7.1%) | 1 (7.1%) | 7 (50%) | 5 (35.7%) |
| Epcoritamab | Supraventricular Tachycardia | 15 | 6 (40%) | 1 (6.7%) | 3 (20%) | 5 (33.3%) |
| Epcoritamab | Atrial Fibrillation | 11 | 5 (45.5%) | 1 (9.1%) | 1 (9.1%) | 4 (36.4%) |
| Epcoritamab | Other Tachyarrhythmia | 16 | 6 (37.5%) | 1 (6.2%) | 3 (18.8%) | 6 (37.5%) |
| Epcoritamab | Fatal CVAE | 71 | 22 (31%) | 14 (19.7%) | 23 (32.4%) | 12 (16.9%) |
| Glofitamab | Hypotension | 10 | 5 (50%) | 1 (10%) | 0 (0%) | 4 (40%) |
| Glofitamab | Shock | 18 | 3 (16.7%) | 9 (50%) | 3 (16.7%) | 3 (16.7%) |
| Glofitamab | Heart Failure | 6 | 0 (0%) | 1 (16.7%) | 1 (16.7%) | 4 (66.7%) |
| Glofitamab | Supraventricular Tachycardia | 8 | 6 (75%) | 1 (12.5%) | 0 (0%) | 1 (12.5%) |
| Glofitamab | Atrial Fibrillation | 5 | 4 (80%) | 1 (20%) | 0 (0%) | 0 (0%) |
| Glofitamab | Other Tachyarrhythmia | 8 | 6 (75%) | 1 (12.5%) | 0 (0%) | 1 (12.5%) |
| Glofitamab | Fatal CVAE | 40 | 5 (12.5%) | 16 (40%) | 4 (10%) | 15 (37.5%) |
| Teclistamab | Hypotension | 14 | 5 (35.7%) | 1 (7.1%) | 1 (7.1%) | 7 (50%) |
| Teclistamab | Shock | 40 | 1 (2.5%) | 23 (57.5%) | 4 (10%) | 12 (30%) |
| Teclistamab | DIC | 3 | 1 (33.3%) | 0 (0%) | 1 (33.3%) | 1 (33.3%) |
| Teclistamab | Heart Failure | 16 | 4 (25%) | 4 (25%) | 2 (12.5%) | 6 (37.5%) |
| Teclistamab | Supraventricular Tachycardia | 11 | 5 (45.5%) | 4 (36.4%) | 0 (0%) | 2 (18.2%) |
| Teclistamab | Atrial Fibrillation | 8 | 4 (50%) | 3 (37.5%) | 0 (0%) | 1 (12.5%) |
| Teclistamab | Other Tachyarrhythmia | 11 | 5 (45.5%) | 4 (36.4%) | 0 (0%) | 2 (18.2%) |
| Teclistamab | Fatal CVAE | 68 | 8 (11.8%) | 32 (47.1%) | 7 (10.3%) | 21 (30.9%) |
| Elranatamab | Hypotension | 15 | 4 (26.7%) | 2 (13.3%) | 0 (0%) | 9 (60%) |
| Elranatamab | Shock | 29 | 3 (10.3%) | 15 (51.7%) | 4 (13.8%) | 7 (24.1%) |
| Elranatamab | DIC | 3 | 1 (33.3%) | 1 (33.3%) | 1 (33.3%) | 0 (0%) |
| Elranatamab | Heart Failure | 14 | 5 (35.7%) | 4 (28.6%) | 1 (7.1%) | 4 (28.6%) |
| Elranatamab | Supraventricular Tachycardia | 10 | 1 (10%) | 2 (20%) | 3 (30%) | 4 (40%) |
| Elranatamab | Atrial Fibrillation | 9 | 1 (11.1%) | 2 (22.2%) | 3 (33.3%) | 3 (33.3%) |
| Elranatamab | Other Tachyarrhythmia | 10 | 1 (10%) | 2 (20%) | 3 (30%) | 4 (40%) |
| Elranatamab | Fatal CVAE | 44 | 7 (15.9%) | 20 (45.5%) | 4 (9.1%) | 13 (29.5%) |
| Talquetamab | Hypotension | 14 | 4 (28.6%) | 2 (14.3%) | 0 (0%) | 8 (57.1%) |
| Talquetamab | Shock | 5 | 0 (0%) | 0 (0%) | 3 (60%) | 2 (40%) |
| Talquetamab | Fatal CVAE | 15 | 2 (13.3%) | 1 (6.7%) | 4 (26.7%) | 8 (53.3%) |
| Limited to drug-outcome combinations with >= 3 events. Independent = absence of both CRS and infection in the same report. | | | | | | |

## S11. Benjamini-Hochberg FDR-Adjusted P-values.

| **Family** | **Drug** | **Outcome** | **aROR** | **P value** | **BH-adjusted P Value** |
| --- | --- | --- | --- | --- | --- |
| Class-level | All BsAbs | Hypotension | 1.61 | 0.0001 | 0.0002 |
| Class-level | All BsAbs | Disseminated intravascular coagulation | 4.35 | 0.0001 | 0.0002 |
| Class-level | All BsAbs | Cytokine release syndrome | 161.19 | 0.0001 | 0.0002 |
| Class-level | All BsAbs | Fatal CVAE | 1.63 | 0.0001 | 0.0002 |
| Disease-class-level | MM BsAbs | Shock | 2.09 | 0.0001 | 0.0003 |
| Disease-class-level | B-ALL BsAbs | Hypotension | 1.49 | 0.0001 | 0.0003 |
| Disease-class-level | MM BsAbs | Hypotension | 1.81 | 0.0001 | 0.0003 |
| Disease-class-level | B-ALL BsAbs | Disseminated intravascular coagulation | 4.53 | 0.0001 | 0.0003 |
| Disease-class-level | MM BsAbs | Disseminated intravascular coagulation | 5.21 | 0.0001 | 0.0003 |
| Disease-class-level | B-ALL BsAbs | Cytokine release syndrome | 30.18 | 0.0001 | 0.0003 |
| Disease-class-level | Lymphoma BsAbs | Cytokine release syndrome | 119.17 | 0.0001 | 0.0003 |
| Disease-class-level | MM BsAbs | Cytokine release syndrome | 146.99 | 0.0001 | 0.0003 |
| Disease-class-level | Lymphoma BsAbs | Fatal CVAE | 1.74 | 0.0001 | 0.0003 |
| Disease-class-level | MM BsAbs | Fatal CVAE | 2.04 | 0.0001 | 0.0003 |
| Disease-class-level | Lymphoma BsAbs | Hypotension | 1.63 | 0.0020 | 0.0053 |
| Disease-class-level | MM BsAbs | Myocarditis | 3.68 | 0.0050 | 0.0122 |
| Disease-class-level | B-ALL BsAbs | Ventricular extrasystole | 4.12 | 0.0100 | 0.0217 |
| Disease-class-level | B-ALL BsAbs | Fatal CVAE | 1.23 | 0.0100 | 0.0217 |
| Drug-level | Teclistamab | Myocarditis | 5.95 | 0.0001 | 0.0005 |
| Drug-level | Talquetamab | Cerebrovascular disease | 40.01 | 0.0001 | 0.0005 |
| Drug-level | Teclistamab | Shock | 2.32 | 0.0001 | 0.0005 |
| Drug-level | Elranatamab | Shock | 2.27 | 0.0001 | 0.0005 |
| Drug-level | Blinatumomab | Hypotension | 1.46 | 0.0001 | 0.0005 |
| Drug-level | Epcoritamab | Hypotension | 2.02 | 0.0001 | 0.0005 |
| Drug-level | Talquetamab | Hypotension | 3.79 | 0.0001 | 0.0005 |
| Drug-level | Blinatumomab | Disseminated intravascular coagulation | 4.41 | 0.0001 | 0.0005 |
| Drug-level | Blinatumomab | Cytokine release syndrome | 87.52 | 0.0001 | 0.0005 |
| Drug-level | Mosunetuzumab | Cytokine release syndrome | 127.57 | 0.0001 | 0.0005 |
| Drug-level | Epcoritamab | Cytokine release syndrome | 267.66 | 0.0001 | 0.0005 |
| Drug-level | Glofitamab | Cytokine release syndrome | 110.91 | 0.0001 | 0.0005 |
| Drug-level | Teclistamab | Cytokine release syndrome | 269.16 | 0.0001 | 0.0005 |
| Drug-level | Elranatamab | Cytokine release syndrome | 295.47 | 0.0001 | 0.0005 |
| Drug-level | Talquetamab | Cytokine release syndrome | 273.25 | 0.0001 | 0.0005 |
| Drug-level | Epcoritamab | Fatal CVAE | 2.06 | 0.0001 | 0.0005 |
| Drug-level | Glofitamab | Fatal CVAE | 1.82 | 0.0001 | 0.0005 |
| Drug-level | Teclistamab | Fatal CVAE | 2.26 | 0.0001 | 0.0005 |
| Drug-level | Elranatamab | Fatal CVAE | 1.90 | 0.0001 | 0.0005 |
| Drug-level | Elranatamab | Disseminated intravascular coagulation | 5.47 | 0.0040 | 0.0164 |
| Drug-level | Glofitamab | Thrombocytopenia | 1.74 | 0.0040 | 0.0164 |
| Drug-level | Talquetamab | Disseminated intravascular coagulation | 7.67 | 0.0050 | 0.0203 |
| Drug-level | Mosunetuzumab | Supraventricular Tachycardia | 2.21 | 0.0100 | 0.0350 |
| Drug-level | Blinatumomab | Ventricular extrasystole | 4.14 | 0.0100 | 0.0350 |
| Drug-level | Mosunetuzumab | Tachyarrhythmia | 2.14 | 0.0100 | 0.0350 |
| Drug-level | Epcoritamab | Disseminated intravascular coagulation | 4.50 | 0.0100 | 0.0350 |
| Drug-level | Epcoritamab | Altered mental status | 1.50 | 0.0100 | 0.0350 |
| Drug-level | Blinatumomab | Fatal CVAE | 1.22 | 0.0100 | 0.0350 |
| Drug-level | Teclistamab | Disseminated intravascular coagulation | 4.07 | 0.0180 | 0.0592 |
| Drug-level | Mosunetuzumab | Atrial fibrillation or flutter | 2.25 | 0.0200 | 0.0627 |
| Drug-level | Elranatamab | Hypotension | 1.70 | 0.0420 | 0.1234 |

Applied to primary analysis signals, stratified by analytic family (class-level, disease-class-level, drug-level). Only signals with raw p<0.05 and aROR>1 shown.

## S12. Firth Penalized Logistic Regression for Sparse Signals (n<10).

| **Drug** | **Outcome** | **N events** | **Standard aROR** | **Firth aROR (95% CI)** | **P-value** |
| --- | --- | --- | --- | --- | --- |
| Elranatamab | Disseminated intravascular coagulation | 3 | 5.47 | 6.65 (1.84 to 16.79) | 0.007 |
| Epcoritamab | Disseminated intravascular coagulation | 3 | 4.50 | 5.39 (1.49 to 13.60) | 0.014 |
| MM BsAbs (class) | Disseminated intravascular coagulation | 8 | 5.21 | 5.67 (2.57 to 10.92) | <0.001 |
| MM BsAbs (class) | Myocarditis | 5 | 3.68 | 4.05 (1.50 to 8.77) | 0.009 |
| Mosunetuzumab | Atrial fibrillation or flutter | 9 | 2.25 | 2.45 (1.18 to 4.44) | 0.018 |
| Talquetamab | Cerebrovascular disease | 1 | 40.01 | 55.68 (6.03 to 226.23) | 0.003 |
| Talquetamab | Disseminated intravascular coagulation | 2 | 7.67 | 10.04 (2.07 to 29.39) | 0.009 |
| Teclistamab | Disseminated intravascular coagulation | 3 | 4.07 | 4.91 (1.35 to 12.55) | 0.020 |
| Teclistamab | Myocarditis | 4 | 5.95 | 7.12 (2.33 to 16.63) | 0.002 |
